# Loss of Cln3 Function in the Social Amoeba *Dictyostelium discoideum* Causes Pleiotropic Effects That Are Rescued by Human CLN3

**DOI:** 10.1371/journal.pone.0110544

**Published:** 2014-10-17

**Authors:** Robert J. Huber, Michael A. Myre, Susan L. Cotman

**Affiliations:** Center for Human Genetic Research, Massachusetts General Hospital, Harvard Medical School, Boston, Massachusetts, United States of America; Université de Genève, Switzerland

## Abstract

The neuronal ceroid lipofuscinoses (NCL) are a group of inherited, severe neurodegenerative disorders also known as Batten disease. Juvenile NCL (JNCL) is caused by recessive loss-of-function mutations in *CLN3*, which encodes a transmembrane protein that regulates endocytic pathway trafficking, though its primary function is not yet known. The social amoeba *Dictyostelium discoideum* is increasingly utilized for neurological disease research and is particularly suited for investigation of protein function in trafficking. Therefore, here we establish new overexpression and knockout *Dictyostelium* cell lines for JNCL research. *Dictyostelium* Cln3 fused to GFP localized to the contractile vacuole system and to compartments of the endocytic pathway. *cln3^−^* cells displayed increased rates of proliferation and an associated reduction in the extracellular levels and cleavage of the autocrine proliferation repressor, AprA. Mid- and late development of *cln3^−^* cells was precocious and *cln3^−^* slugs displayed increased migration. Expression of either *Dictyostelium* Cln3 or human CLN3 in *cln3^−^* cells suppressed the precocious development and aberrant slug migration, which were also suppressed by calcium chelation. Taken together, our results show that Cln3 is a pleiotropic protein that negatively regulates proliferation and development in *Dictyostelium*. This new model system, which allows for the study of Cln3 function in both single cells and a multicellular organism, together with the observation that expression of human CLN3 restores abnormalities in *Dictyostelium cln3^−^* cells, strongly supports the use of this new model for JNCL research.

## Introduction

The neuronal ceroid lipofuscinoses (NCL) are a group of inherited, severe neurodegenerative disorders also known as Batten disease [1]. At the cellular level, NCL disorders characteristically display aberrant lysosomal function and an excessive accumulation of lipofuscin in neurons and other cell types [2], [3]. Clinical manifestations include vision loss, seizures, the progressive loss of motor function and psychological ability, and a reduced lifespan [4]. Recent evidence also points to pathology outside of the central nervous system, more specifically the cardiac and immune systems [5]–[8]. North American and Northern European populations have the highest rates of incidence, however the NCL disorders have a worldwide distribution with varying incidence rates depending on the region (1∶14000 to 1∶100000) [9]. Currently there are no effective treatments or cure for NCL disorders.

Juvenile NCL (JNCL), the most common subtype of NCL, occurs due to recessive mutations in the *CLN3* gene with the majority of JNCL patients carrying a ∼1-kb genomic deletion spanning exons 7 and 8 [10]. Indel, missense, nonsense, and splice site mutations have also been documented in JNCL patients [11], [12]. In mammals, *CLN3* encodes a 438 amino acid multi-pass transmembrane protein (CLN3/battenin; ceroid-lipofuscinosis, neuronal 3) that is primarily found in endosomes and lysosomes with evidence that it may also traffic to other subcellular membranes [3], [13], [14]. In neurons, CLN3 may be important for events localized at the synapse [15]. Evidence from yeast and mouse models independently suggests that CLN3 may function in lysosomal pH homeostasis, endocytic trafficking, and autophagy [16]–[20]. Despite substantial research efforts using a variety of systems, the precise function of CLN3 remains unclear [21].

A new, unexplored approach to studying CLN3 function involves the use of the social amoeba *Dictyostelium discoideum*, which has been selected by the National Institutes of Health as a model organism for biomedical and human disease research. This genetically tractable model eukaryote is being used successfully to study the function of genes linked to neurodegenerative disorders and is particularly suited to modeling human lysosomal and trafficking diseases [22]–[29]. *Dictyostelium* is a soil microbe that undergoes an asexual life cycle composed of a growth phase in which single cells grow and divide mitotically as they feed on bacteria and a multicellular developmental stage that is induced upon starvation. During the early stages of *Dictyostelium* development, the starving population of cells secretes cAMP in a pulsatile manner, which serves to attract individual cells chemotactically to form a multicellular aggregate also referred to as a mound. After a series of morphological changes, the mound develops into a slug-like structure that is capable of both photo- and thermotaxis. When conditions are suitable, the slug, composed of predominantly two cell types (i.e., pre-stalk and pre-spore), completes the life cycle by forming a fruiting body comprised of a mass of spores supported by a stalk of dead cells. When a food source becomes available, the spores germinate allowing the amoeba to re-start the life cycle. Thus, *Dictyostelium* serves as a valuable system for studying a variety of cell and developmental processes [30]–[32].

Understanding the normal function of CLN3 is a key step in designing targeted therapies for JNCL. Therefore, in this study, we have established new tools for research into CLN3 function by generating a Cln3-deficient *Dictyostelium* mutant by targeted homologous recombination and introducing GFP-tagged *Dictyostelium* Cln3 and human CLN3 into *Dictyostelium* cells. Assessment of the knockout and overexpression cells during growth and development strongly indicates that the function of CLN3 is conserved from *Dictyostelium* to human. Furthermore, our results strongly support a key role for CLN3 in regulating the endocytic pathway and calcium-dependent developmental events.

## Materials and Methods

### Cells and chemicals

AX3 and *cln3^−^* cells were grown and maintained at room temperature on SM agar with *Klebsiella aerogenes* and in HL5 medium supplemented with ampicillin (100 µg/ml) and streptomycin sulfate (300 µg/ml). *cln3^−^* cells also required blasticidin S hydrochloride (10 µg/ml), while strains carrying the extrachromosomal vector pTX-GFP required G418 (10 µg/ml) [33]. HL5, FM minimal medium, and low fluorescence HL5 were purchased from ForMedium (Hunstanton, Norfolk, UK). The QIAquick PCR Purification Kit, QIAquick Gel Extraction Kit, and QIAprep Spin Miniprep Kit were used for all PCR purifications, gel extractions, and plasmid isolations, respectively, and were all purchased from Qiagen Incorporated (Valencia, CA, USA). Restriction enzymes were purchased from New England BioLabs Incorporated (Ipswich, MA). All primers were purchased from Integrated DNA Technologies Incorporated (Coralville, IA, USA). EGTA and FITC-dextran were purchased from Sigma-Aldrich (St. Louis, MO, USA). Mouse monoclonal anti-p80 was purchased from the Developmental Studies Hybridoma Bank (University of Iowa, Iowa City, IA, USA).

### Axenic growth and pinocytosis

Cells in the mid-log phase of growth (1–5×10^6^ cells/ml) were diluted to 1–2×10^5^ cells/ml in fresh HL5 or FM and incubated at 22°C and 150 rpm. Cell concentrations were measured every 24 hours over a 120- or 144-hour growth period with a hemocytometer. Pinocytosis assays were performed as previously described [34]. Briefly, AX3 and *cln3^−^* cells (5×10^6^ cells/ml) were grown in HL5. FITC-dextran (70,000 M_r_, 100 µl of a 20 mg/ml solution) was added to a 5-ml cell suspension, which was then incubated for 90 minutes at room temperature and 150 rpm. Equal volumes of cells (500 µl) were harvested at the indicated times, washed 2 times with ice-cold Sorenson’s buffer (2 mM Na_2_HPO_4_, 14.6 mM KH_2_PO_4_, pH 6.0), and then lysed with 1 ml of buffer containing 50 mM Na_2_HPO_4 _pH 9.3 and 0.2% Triton-X. Lysates were placed in black 96-well plates and fluorescence was measured with a Molecular Devices SpectraMax M2 Multi-Mode Microplate Reader (excitation 470, emission 515). For axenic growth and pinocytosis assays, statistical significance was assessed in GraphPad Prism 5 (GraphPad Software Incorporated, La Jolla, CA, USA) using two-way ANOVA followed by Bonferroni post-hoc analysis. A p-value<0.05 was considered significant (i.e., n = # of independent cell cultures; see relevant Figure legends for additional details). For experiments assessing the effect of *cln3* knockout on the intra- and extracellular levels of AprA and CfaD, AX3 and *cln3^−^* cells grown axenically in HL5 (as described above) were harvested and lysed after 48 and 72 hours of growth. At each of these time points, cells from 15 ml of culture were also spun down and conditioned media was collected and filtered through a 0.45 µm filter unit. Samples were standardized by loading volumes of conditioned media according to cell number (i.e., media from 100000 cells). Whole cell lysates and samples of conditioned media were separated by SDS-PAGE and analyzed by western blotting.

### Development

Development assays were performed as previously described [35]. Briefly, cells grown in HL5 were harvested in the mid-log phase of growth (1–5×10^6^ cells/ml) and washed two times with ice-cold KK2 phosphate buffer (2.2 g/L KH_2_PO_4_, 0.7 g/L K_2_HPO_4_, pH 6.5). Washed cells (3×10^7^ cells/ml) were deposited in four individual cell droplets (25 µl each droplet) on black, gridded, cellulose filters (0.45 mm pore size) (EMD Millipore Corporation, Billerica, MA, USA) overlaid on four Whatman #3 cellulose filters (EMD Millipore Corporation, Billerica, MA, USA) pre-soaked in KK2 buffer. Cells were maintained in the dark in a humidity chamber at room temperature. Structures were viewed and photographed at the indicated times with a Nikon SMZ800 microscope (Nikon Instruments Incorporated, Melville, NY, USA) equipped with a SPOT Insight color camera 3.2.0 (Diagnostic Instruments Incorporated, Sterling Heights, MI, USA). Images were captured with SPOT for Windows (Diagnostic Instruments Incorporated, Sterling Heights, MI, USA). For each independent experiment, developmental phenotypes were scored for each cell droplet (i.e., 4 total) and then averaged to obtain a mean value for that experiment (i.e., n = # of independent experiments; see relevant Figure legends for additional details). Statistical significance was assessed in GraphPad Prism 5 (GraphPad Software Incorporated, La Jolla, CA, USA). Data that satisfied parametric requirements were analyzed using one-way ANOVA followed by the Bonferroni multiple comparison test. Non-parametric data were analyzed using the Kruskal-Wallis test followed by the Dunn multiple comparison test. A p-value<0.05 was considered significant. See relevant Figure legends for additional details.

### Live cell imaging, fxation, and immunolocalization

Cells were viewed live in 6-well dishes containing water or low fluorescence HL5. Fixation in ultra-cold methanol (for cells probed with anti-VatM or anti-Rh50) or 4% paraformaldehyde (for cells probed with anti-p80) followed by immunolocalization, were performed as previously described [36], [37]. Prior to fixation, cells were grown overnight on coverslips in low fluorescence HL5. The following primary and secondary antibodies were used; rabbit polyclonal anti-GFP (1∶1000) (Life Technologies Incorporated, Carlsbad, CA, USA), mouse monoclonal anti-GFP (1∶50) (Santa Cruz Biotechnology Inc., Santa Cruz, CA, USA), mouse monoclonal anti-VatM (1∶10–1∶25) [38], rabbit polyclonal anti-Rh50 (1∶1500–1∶2000) [39], mouse monoclonal anti-p80 (1∶50) [40], donkey anti-rabbit Alexa Fluor 488, donkey anti-rabbit Alexa Fluor 555, donkey anti-mouse Alexa Fluor 488, and donkey anti-mouse Alexa Fluor 555 (1∶50–1∶100) (Life Technologies Incorporated, Carlsbad, CA, USA). Coverslips were mounted on slides with Prolong Gold anti-fade reagent with DAPI (Life Technologies Incorporated, Carlsbad, CA, USA) and sealed with nail polish. Live cells were viewed with a Nikon Eclipse TE2000-U microscope equipped with Nikon Digital Sight DS-Qi1Mc and Nikon Digital Sight DS-Fi1 digital cameras (Nikon Instruments Incorporated, Melville, NY, USA). Fixed cells were imaged either with a Leica SP5 AOBS scanning laser confocal microscope (Leica Microsystems, Buffalo Grove, IL, USA) or a Zeiss Axioskop2 mot plus epifluorescence microscope equipped with a Zeiss AxioCam MRm digital camera (Carl Zeiss Microscopy LLC, Thornwood, NY, USA). For confocal analysis, the separate channels were imaged using sequential scanning mode and z-sections were taken with a pinhole setting of 1 airy unit (AU). Separate channel and overlay (i.e., merge) images were exported from the Leica imaging software (LAS AF), or from the Zeiss AxioVision imaging software (version 4.6.3), as.tif files and opened into Adobe Photoshop CS5 for compilation of figures. For epifluorescence images, the merge of the separate channel images was produced using ImageJ/Fiji software. If minor brightness and contrast adjustments were necessary, these were made in Photoshop uniformly for each set of images of a given co-stain combination.

### SDS-PAGE and western blotting

Cells were lysed with a buffer containing 50 mM Tris–HCl pH 8.0, 150 mM sodium chloride, 0.5% NP-40, 5 mM EDTA, 10 mM sodium fluoride, 1 mM sodium orthovanadate, and a protease inhibitor cocktail tablet (Roche Diagnostics Corporation, Indianapolis, IN, USA). Proteins were separated by SDS-PAGE and analyzed by western blotting with mouse monoclonal anti-tubulin (1∶1000) (12G10, Developmental Studies Hybridoma Bank, The University of Iowa, IA, USA), mouse monoclonal anti-actin (1∶1000), mouse monoclonal anti-GFP (1∶1000) (Santa Cruz Biotechnology Inc., Santa Cruz, CA, USA), rabbit polyclonal anti-AprA (1∶1000) [41], and rabbit polyclonal anti-CfaD (1∶1000) [42]. Immunoblots were digitally scanned using a GS800 Calibrated Densitometer scanner and Quantity One software (Bio-Rad Laboratories Incorporated, Hercules, CA, USA). Identified bands were quantified with ImageJ/Fiji and levels were normalized to ß-actin levels. Results were pooled from four independent experiments, each with at least two technical replicates. Statistical significance was determined using a one-sample t-test (mean, 100; two-tailed). A p-value<0.05 was considered significant.

### Bioinformatic and phylogenetic analysis

Sequence alignments between *Dictyostelium* Cln3 and human CLN3 were performed using the dictyBase BLAST server (http://www.dictybase.org/tools/blast). For phylogenetic analyses, the amino acid sequence of *Dictyostelium* Cln3 was inputted into the NCBI BLASTp server. Amino acid sequences for significant hits corresponding to CLN3 orthologs from 20 different organisms (i.e., mammals and NIH model systems) were obtained and aligned using ClustalX version 1.83. Neighbor-Joining trees were created using ClustalX version 1.83 and PAUP version 4.0 (Sinauer Associates Incorporated Publishers, Sunderland, MA, USA) and viewed using TreeView version 1.6.6.

### Gene knockout and validation

Targeted disruption of the c*ln3* gene in *Dictyostelium discoideum* was accomplished using an approach that has been previously described [26]. Targeting arms were amplified by PCR using the Expand High-Fidelity PCR System (Roche Diagnostics Corporation, Indianapolis, IN, USA) and cloned into vector pLPBLP, which knocked out the gene of interest by homologous recombination and introduced a blasticidin resistance (*bsr*) cassette [43]. The 5′ targeting arm was amplified using the following primers, which incorporated *Kpn*I and *Hin*dIII sites (underlined) to facilitate directional cloning into pLPBLP; 5′-GGTACCTCTTTATACTATATATTATACCTCCTTCTC-3′ (forward) and 5′-AAGCTTCATCTTGAAACTAAACCAAATGCAATATTTGC-3′ (reverse). The 3′ targeting arm was amplified using the following primers, which incorporated *Pst*I and *Spe*I (underlined) to facilitate directional cloning into pLPBLP; 5′-CTGCAGAAAACAAAGATATATTCGTTGTGCACG-3′ (forward) and 5′- ACTAGTATGAAGAATCAGTTTTTGGAACCTCAGAG-3′ (reverse). AX3 cells were electroporated with 10 µg of linearized gene-targeting DNA. 96 colonies resistant to blasticidin S hydrochloride (10 µg/ml) were collected and replica-plated into a 96-well plate. Genomic DNA was extracted using the DNeasy Blood and Tissue Kit (Qiagen Incorporated, Valencia, CA, USA) and targeted gene disruption was validated by nine PCR reactions using a combination of primers (File S1, Table S1). PCR analysis identified eight positive clones that all showed a similar growth phenotype (discussed in Results). Two of these clones were further analyzed by Southern blotting. Genomic DNA from each clone was isolated and digested overnight with *Hin*dIII at 37°C, separated by agarose gel electrophoresis, and transferred to positively charged nylon membranes by capillary transfer. Blots were hybridized with a DIG-labelled probe corresponding to the entire sequence of the *bsr* gene using the PCR DIG Probe Synthesis Kit and the DIG High Prime DNA Labeling and Detection Starter Kit II according to the manufacturer’s instructions (Roche Diagnostics Corporation, Indianapolis, IN, USA). The *bsr* gene was amplified from pLPBLP using the following primers; 5′-ATGGATCAATTTAACATTTCTCAAC-3′ (forward) and 5′-TTAATTTCGGGTATATTTGAGTGG-3′ (reverse). Based on the position of *Hin*dIII cut sites in the *Dictyostelium* genome, a single 2746 bp fragment was expected on Southern blots probed with the DIG-labelled *bsr* probe (www.dictybase.org). A ∼2750 bp fragment was detected in both clones however one of the clones also contained an unexpected ∼6600 bp fragment. Since this implied an unintended and possibly complex integration event, we chose to work with the clone containing the single ∼2750 bp fragment. We designated this clone as the *cln3* knockout strain and used these cells in all subsequent analyses.

### Construction of GFP expression constructs and cell lines

Vector pTX-GFP, which incorporates an N-terminal GFP tag, was used to generate all GFP-fusion protein constructs [33]. Full-length *Dictyostelium cln3* was amplified from cDNA using the following primers, which incorporated *Sac*I and *Xho*I sites (underlined) to facilitate directional cloning into pTX-GFP; 5′-GAGCTCATGGGAAAGGATTATACATT-3′ (forward) and 5′-CTCGAGTTATGTTGAGGATGAAGAAT-3′ (reverse). Full-length human *CLN3* was amplified from cDNA using the following primers, which also incorporated *Sac*I and *Xho*I sites (underlined); 5′-GAACTTGAGCTCATGGGAGGCTGTG-3′ (forward) and 5′-TAATCCCTCGAGTCAGGAGAGCTGGC-3′ (reverse). To facilitate the expression of *Dictyostelium* GFP-Cln3 and human GFP-CLN3 at close to endogenous levels, the *act15* promoter and the first 11 codons of the GFP open reading frame, which contained the initiation methionine and an amino-terminal 8x histidine tag, was removed from pTX-GFP by digesting the plasmid with *Sal*I and *Kpn*I. Three fragments containing DNA from the non-coding region directly upstream of *cln3* were amplified from AX3 gDNA using primers cln3_up_elem_F1, cln3_up_elem_F2, cln3_up_elem_F3, and cln3_up_elem_R1 (File S1, Table S1). Forward primers incorporated *Sal*I restriction sites and reverse primers incorporated *Kpn*I restriction sites to facilitate directional cloning into pTX-GFP. The longest fragment (i.e., upstream element 1) spanned the entire region upstream of the *cln3* start site up to the end of the preceding gene (File S1, Fig. S1). The other two fragments (i.e., upstream elements 2 and 3) spanned regions within upstream element 1 up to the *cln3* start site. The three upstream elements, which also included the first 36 base pairs (12 codons) of the *cln3* open reading frame, were then separately cloned into pTX-GFP upstream and in-frame with the GFP open reading frame. All constructs were validated by agarose gel electrophoresis and DNA sequencing (CHGR Genotyping Resource, Genomics Core Facility, Massachusetts General Hospital, Boston, MA, USA). The ability of each *cln3* upstream element to drive GFP expression in AX3 cells was verified by western blotting (File S1, Fig. S1). Since upstream element 1 was the strongest driver of gene expression (File S1, Fig. S1), we used this fragment of DNA, hereafter referred to as ‘cln3 upstream element’, to drive gene expression in our modified version of pTX-GFP (i.e., *act15* promoter removed).

## Results

### Sequence analysis of *Dictyostelium* Cln3

**Figure 1:**
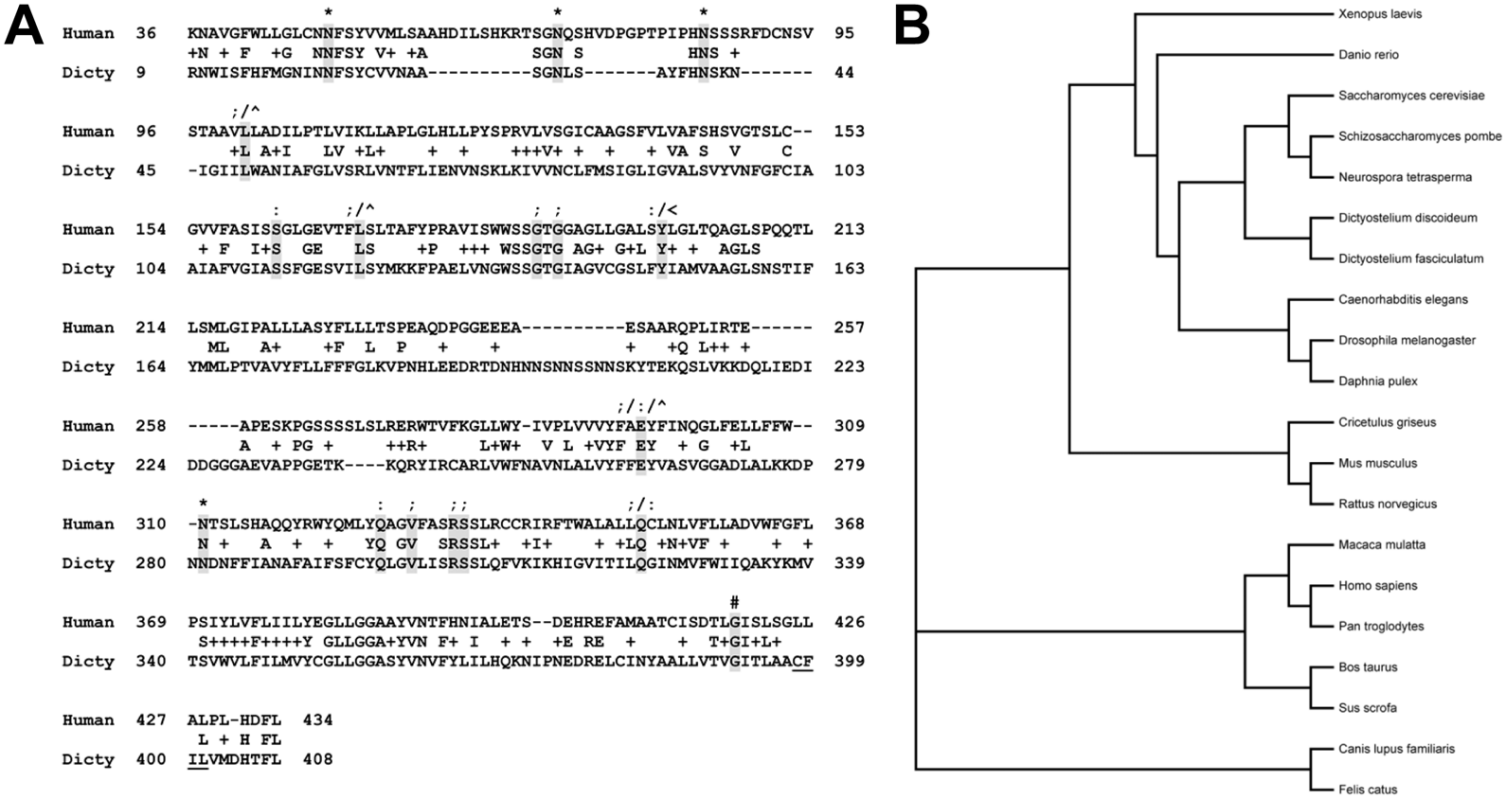
Bioinformatic analysis of *Dictyostelium* Cln3. (A) Alignment of human CLN3 and the *Dictyostelium* ortholog. The following residues are conserved; N-linked glycosylation sites (*), sites of missense point mutations (;), sites of nonsense point mutations (:), target for myristoylation (#), sites that when mutated cause a slower disease progression in compound heterozygosity with the common 1.02 kb deletion mutation (∧), sites that when mutated cause a slower disease progression in homozygosity (<) [10], [13], [85]–[87]. *Dictyostelium* Cln3 also contains a putative prenylation motif (i.e., CFIL; underlined). (B) Phylogenetic tree showing the relationship of *Dictyostelium* Cln3 to CLN3 orthologs from 20 different organisms (i.e., mammals and NIH model systems).

The 438 amino acid sequence of human CLN3 was inputted into the dictyBase BLASTp server (http://www.dictybase.org/tools/blast). The highest match was a 421 amino acid protein (Cln3; DDB_G0291157). There were 117 exact matches (27% identical) and 197 positive matches (46% similar) within a 429 amino acid region of similarity (Fig. 1A). In comparison, the CLN3 homolog in *Saccharomyces cerevisiae*, Btn1p, is 38% identical and 49% similar to the human protein, while the *Schizosaccharomyces pombe* homolog is 32% identical and 47% similar. However, the CLN3 homologs in yeast are comparatively smaller than *Dictyostelium* Cln3 (408 aa and 396 aa vs. 421 aa). Residues that are myristoylated or glycosylated in human CLN3 are conserved in *Dictyostelium* Cln3 and a putative prenylation motif near the C-terminus of the protein (i.e., 398-CFIL-401) is present, although it does not precisely align with the prenylation motif in the human protein, which is found at the end of the protein (i.e., 435-CQLS-438) (Fig. 1A). Importantly, point mutations (missense and nonsense) documented from JNCL patients are highly conserved in the *Dictyostelium* ortholog (Fig. 1A). Together, these similarities indicate that the function of the protein is likely conserved from *Dictyostelium* to human. A phylogenetic tree showing the relationship of *Dictyostelium* Cln3 to CLN3 orthologs from 20 different organisms of interest (i.e., NIH model systems and mammals) firmly places *Dictyostelium* Cln3 within the CLN3 family of proteins (Fig. 1B).

### *Dictyostelium* Cln3 fused to GFP localizes to the contractile vacuole network and to vesicles of the endocytic pathway

**Figure 2:**
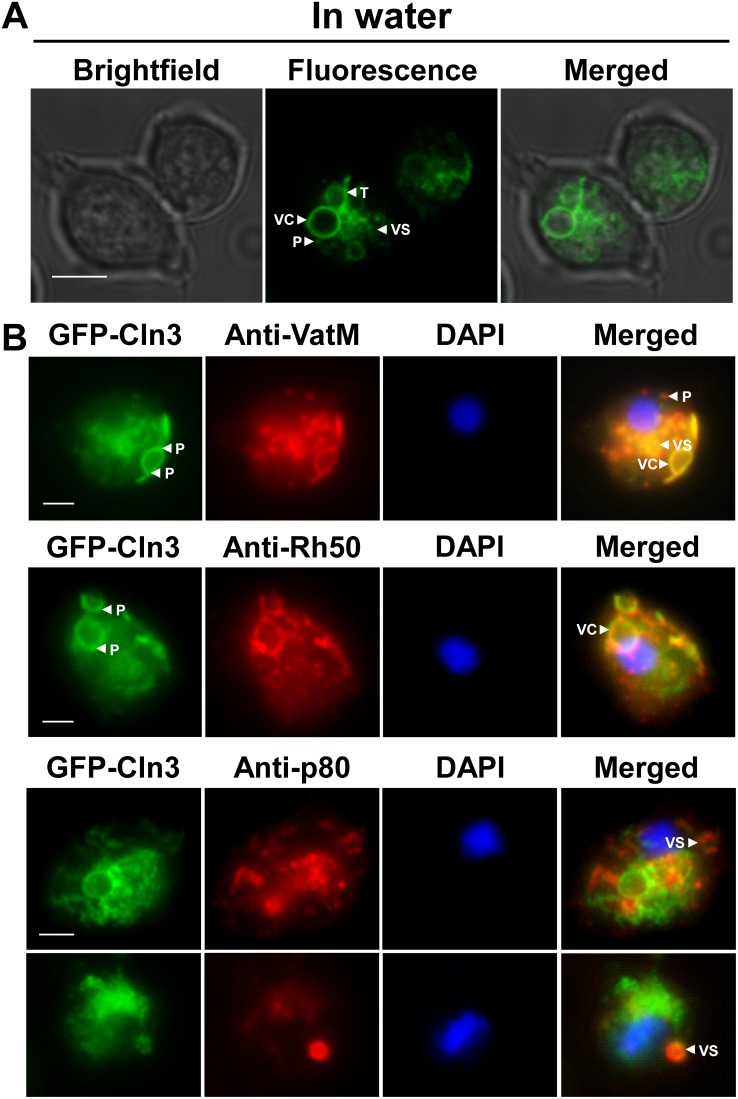
Intracellular localization of *Dictyostelium* GFP-Cln3 using epifluorescence microscopy. (A) AX3 cells overexpressing GFP-Cln3 imaged live in water. Scale bar = 5 µm. (B) AX3 cells overexpressing GFP-Cln3 were fixed in either ultra-cold methanol (for VatM and Rh50 immunostaining) or 4% paraformaldehyde (for p80 immunostaining) and then probed with anti-VatM, anti-Rh50, or anti-p80, followed by the appropriate secondary antibody linked to Alexa 555. Cells were stained with DAPI to reveal nuclei (blue). Images were merged with ImageJ/Fiji. VC, vacuolar-shaped structures; VS, cytoplasmic vesicles; T, tubular-like structures within the cytoplasm; P, punctate distributions within the cytoplasm. Scale bars (B, C) = 2.5 µm.

To gain insight into the function of the CLN3 ortholog in *Dictyostelium*, we transformed AX3 cells with a vector that expressed *Dictyostelium* Cln3 fused to GFP. We chose to place the GFP tag on the N-terminus since a previous study has reported the mis-localization of CLN3 tagged with C-terminal GFP, presumably due to the masking of the prenylation motif [44]. Protein expression was verified by western blotting and a thorough discussion and analysis of the banding pattern is provided in the supporting information (File S1, Fig. S2). In live AX3 cells incubated in water, *Dictyostelium* GFP-Cln3 localized to the membranes of vacuolar-shaped structures and small cytoplasmic vesicles, to tubular-like structures within the cytoplasm, and as punctate distributions within the cytoplasm (Fig. 2A). Time-lapse video microscopy of these cells showed multiple vacuoles undergoing dynamic events of expansion and contraction (File S1, Fig. S3). In free-living amoebae and protozoa, the contractile vacuole (CV) system acts as an osmoregulatory organelle that controls the intracellular water balance by collecting and expelling excess water out of the cell. In *Dictyostelium*, the CV system consists of tubules and vacuoles that function to collect and expel excess water, respectively [45]. Based on our initial observations of *Dictyostelium* GFP-Cln3 localization in AX3 cells, we next fixed and probed cells expressing GFP-Cln3 with antibodies directed against two established *Dictyostelium* CV system markers, the V-ATPase membrane subunit (VatM) and the rhesus-like glycoprotein Rh50 [38], [39]. VatM generates an acidic environment in several intracellular compartments and is found in both the CV and endosomal systems, however it is enriched ∼10-fold in the CV system, while Rh50 is more specific to the CV system [38], [39], [46], [47]. GFP-Cln3 was found to strongly localize to both VatM- and Rh50-positive compartments (Fig. 2B). Interestingly, much like VatM, GFP-Cln3 localized to both small cytoplasmic vesicles and at distinct punctate distributions within the cytoplasm (Fig. 2B). GFP-Cln3 was also observed to localize as punctate clusters on the vacuolar membrane (Fig. 2B). Since localization of GFP-Cln3 was observed on the smaller, VatM-positive punctate distributions, we also assessed localization of GFP-Cln3 to p80-positive compartments. The p80 protein localizes to late endosomes during *Dictyostelium* growth [40]. Although GFP-Cln3 localized primarily to the vacuoles of the CV system (Fig. 2A,B), which were unstained by the p80 antibody, we did observe GFP-Cln3 localization on the membranes of a subset of small cytoplasmic vesicles that were also stained by the p80 antibody (Fig. 2B).

**Figure 3:**
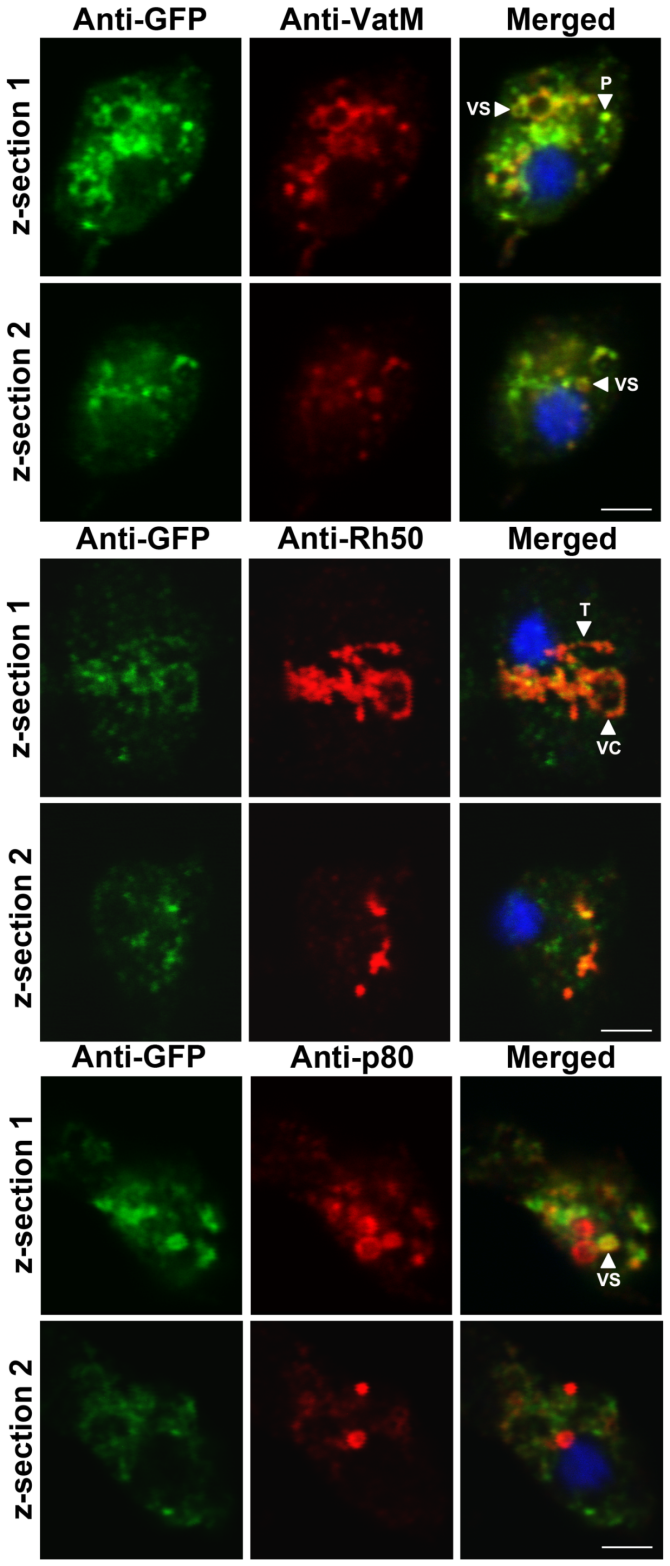
Intracellular localization of *Dictyostelium* GFP-Cln3 using confocal microscopy. AX3 cells overexpressing GFP-Cln3 were fixed in either ultra-cold methanol (for VatM and Rh50 immunostaining) or 4% paraformaldehyde (for p80 immunostaining) and then probed with anti-GFP (rabbit polyclonal anti-GFP for anti-VatM and anti-p80 co-staining and mouse monoclonal anti-GFP for anti-Rh50 co-staining) followed by anti-rabbit or anti-mouse Alexa 488. Cells were then probed with one of anti-VatM, anti-Rh50, or anti-p80 followed by the appropriate secondary antibody linked to Alexa 555. Two z-sections are shown for each cell. Z-sections 1 and 2 are approximately 1 µm and 3 µm, respectively, from the bottom of each cell. VC, vacuolar-shaped structures; VS, cytoplasmic vesicles; T, tubular-like structures within the cytoplasm; P, punctate distributions within the cytoplasm. Scale bars = 2.5 µm.

To further support the localization of *Dictyostelium* GFP-Cln3 to VatM-, Rh50-, and p80-positive subcellular compartments, we analyzed the localization of GFP-Cln3 using immunofluorescence and confocal microscopy. Across multiple z-sections of the amoeboid *Dictyostelium* cells, GFP-Cln3 localized to VatM-positive vesicles and punctate distributions, Rh50-positive tubules and vacuolar-shaped structures, and a subset of p80-positive vesicles (Fig. 3). Taken together, our data strongly suggest that Cln3 localizes to both the CV and endocytic systems in *Dictyostelium*.

### *Cln3^−^* cells show enhanced rates of proliferation and increased intracellular accumulation of FITC-dextran

**Figure 4:**
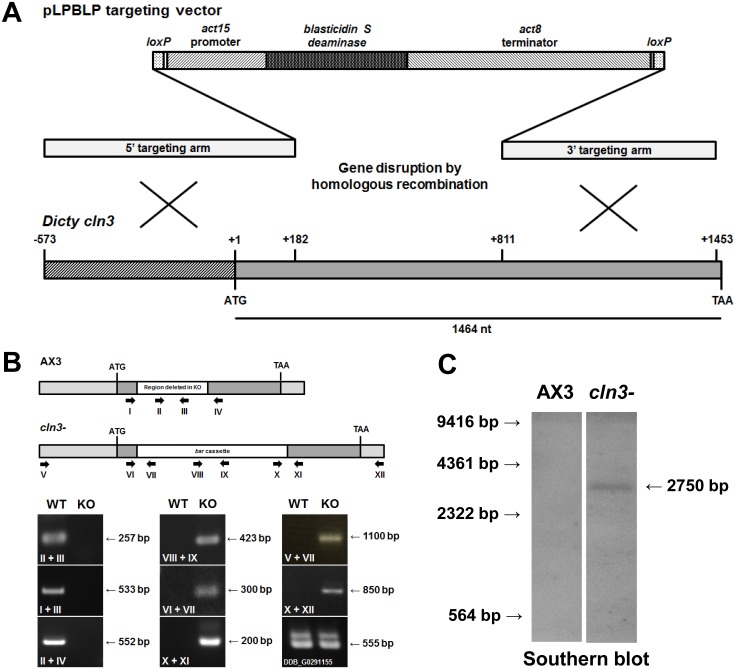
Generation of a *Dictyostelium cln3* knockout mutant. (A) Creation of a *Dictyostelium cln3* knockout mutant by homologous recombination. The pLPBLP targeting vector and sites of recombination are shown. (B) Validation of *cln3* knockout by PCR analysis. Primers are denoted by Roman numerals and arrows. The *Dictyostelium* gene denoted DDB_G0291155 lies downstream of *cln3* and was amplified to confirm that the insertion of the *bsr* cassette did not affect this gene. (C) Validation of *cln3* knockout by Southern blotting. DNA ladder (in bp) is shown to the left of the blot.

To further study the function of Cln3 in *Dictyostelium*, a *cln3* knockout mutant was generated by targeted homologous recombination, which deleted the entire region spanning amino acids 61–421 (Fig. 4A–C). RNA-Seq data shows that expression of *cln3* mRNA decreases by ∼30% during the first 4 hours of development, but then increases dramatically during the next 8 hours (i.e., ∼8-fold increase), with expression peaking after 12 hours of development [48]. Expression decreases slightly between 12 and 20 hours (∼15% decrease), but overall remains high during the mid- to late stages of *Dictyostelium* development.

**Figure 5:**
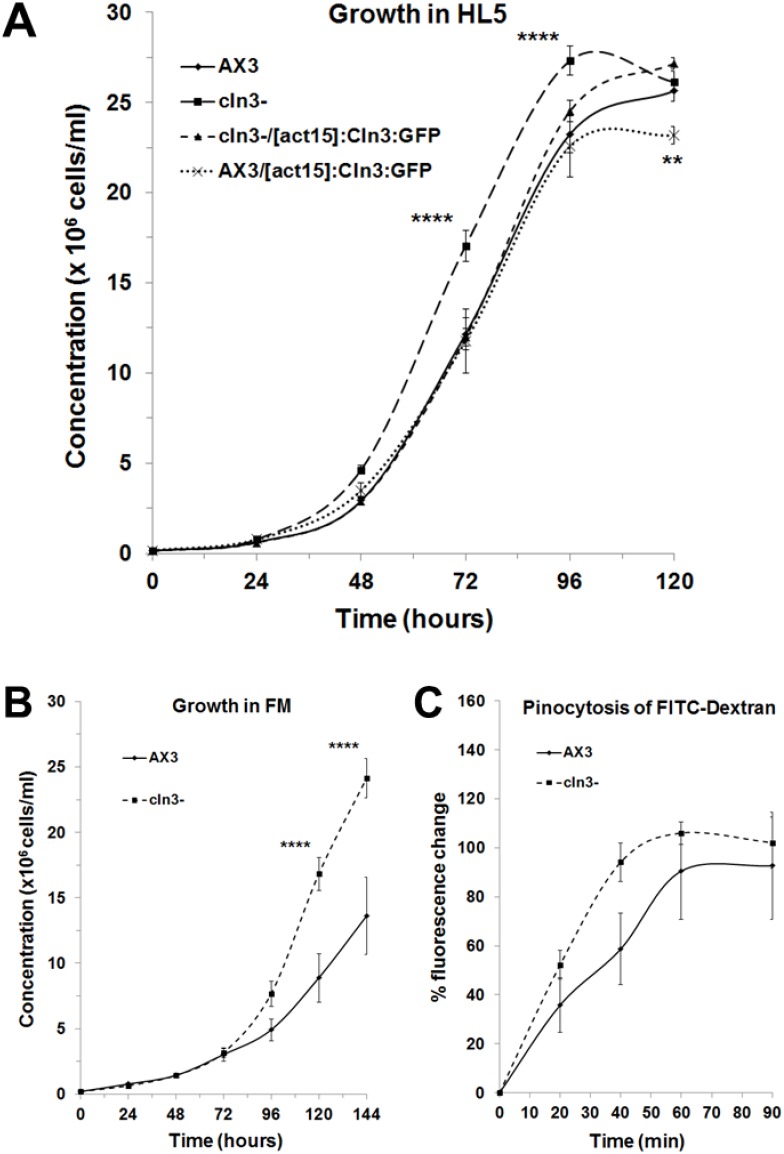
Effect of *cln3* knockout on cell proliferation and pinocytosis. (A) Axenic growth of AX3 *cln3^−^, cln3^−^*/[*act15*]:Cln3:GFP, and AX3/[*act15*]:Cln3:GFP cells in HL5 medium. Data presented as mean concentration (×10^6^ cells/ml) ± s.e.m (n = 10–20). (B) Axenic growth of AX3 and *cln3^−^* cells in FM medium. Data presented as mean concentration (×10^6^ cells/ml) ± s.e.m (n = 8). (C) Effect of *cln3* knockout on the intracellular accumulation of FITC-dextran. Data presented as mean % fluorescence change ± s.e.m (n = 10). Statistical significance was assessed using two-way ANOVA followed by Bonferroni post-hoc analysis. Two-way ANOVA revealed a significant effect of genotype on the growth curves shown in panels A and B (p<0.001 and p<0.01, respectively). **p-value<0.01 and ****p-value<0.0001 vs. AX3 as determined from Bonferroni post-hoc analysis at the indicated time points.

Since growth is a major phase of the *Dictyostelium* life cycle, we first assessed the effect of Cln3 deficiency on the rate of cell proliferation in axenic media. In HL5, *cln3^−^* cells proliferated at a significantly enhanced rate compared to parental AX3 cells (genotype effect, two-way ANOVA, p<0.001) (Fig. 5A). However, no significant difference was observed between the highest densities attained by both strains after 120 hours of growth (Fig. 5A). Since we were able to successfully overexpress *Dictyostelium* GFP-Cln3 in AX3 and *cln3^−^* cells, we next assessed the ability of GFP-Cln3 to alter the enhanced rate of proliferation of *cln3^−^* cells and the effect of GFP-Cln3 overexpression on AX3 cell proliferation. GFP-Cln3 overexpression significantly suppressed the enhanced proliferation of *cln3^−^* cells to levels observed in AX3 cells (Fig. 5A). Overexpression of GFP-Cln3 in AX3 cells had no significant effect on cell proliferation however these cells reached a significantly lower final density after 120 hours when compared to all other strains (Fig. 5A). Based on these results, we then assessed the growth of *cln3^−^* cells in FM minimal media to determine whether limiting available nutrients would suppress the enhanced growth rate. When grown in FM, cells of both strains proliferated at a reduced rate compared to growth in HL5 (Fig. 5A, B). We did not detect any significant differences in the growth rates of AX3 and *cln3^−^* cells during the first 96 hours of growth in FM (Fig. 5B). However, at the 120- and 144-hour time points, *cln3^−^* cells were at a significantly higher density than AX3 cells, and the genotype was found to have a significant effect on the overall growth curve, as determined by two-way ANOVA (p<0.01) (Fig. 5B).

Since pinocytosis is required for the growth of *Dictyostelium* cells in liquid media, we used a well-established assay to assess whether this process was dysregulated in *cln3^−^* cells. AX3 and *cln3^−^* cells were incubated with FITC-dextran, and the amount of intracellular fluorescence was measured at specific time intervals over a 90-minute incubation period. At the 40-minute time point, the intracellular fluorescence was relatively higher (∼50%) in *cln3^−^* cells compared to AX3 cells (Fig. 5C). However, two-way ANOVA analysis of the pinocytic uptake of FITC-dextran over the entire 90-minute incubation period did not indicate a statistically significant genotype effect (p>0.05) (Fig. 5C). Although one of the pathological hallmarks of JNCL is the accumulation of lysosomal storage material in neurons and other cell types [2], [3], we were unable to observe any autofluorescent material in *cln3^−^* cells during growth (unpublished data).

### Cln3 deficiency negatively affects the secretion and cleavage of autocrine proliferation repressor a during growth

**Figure 6:**
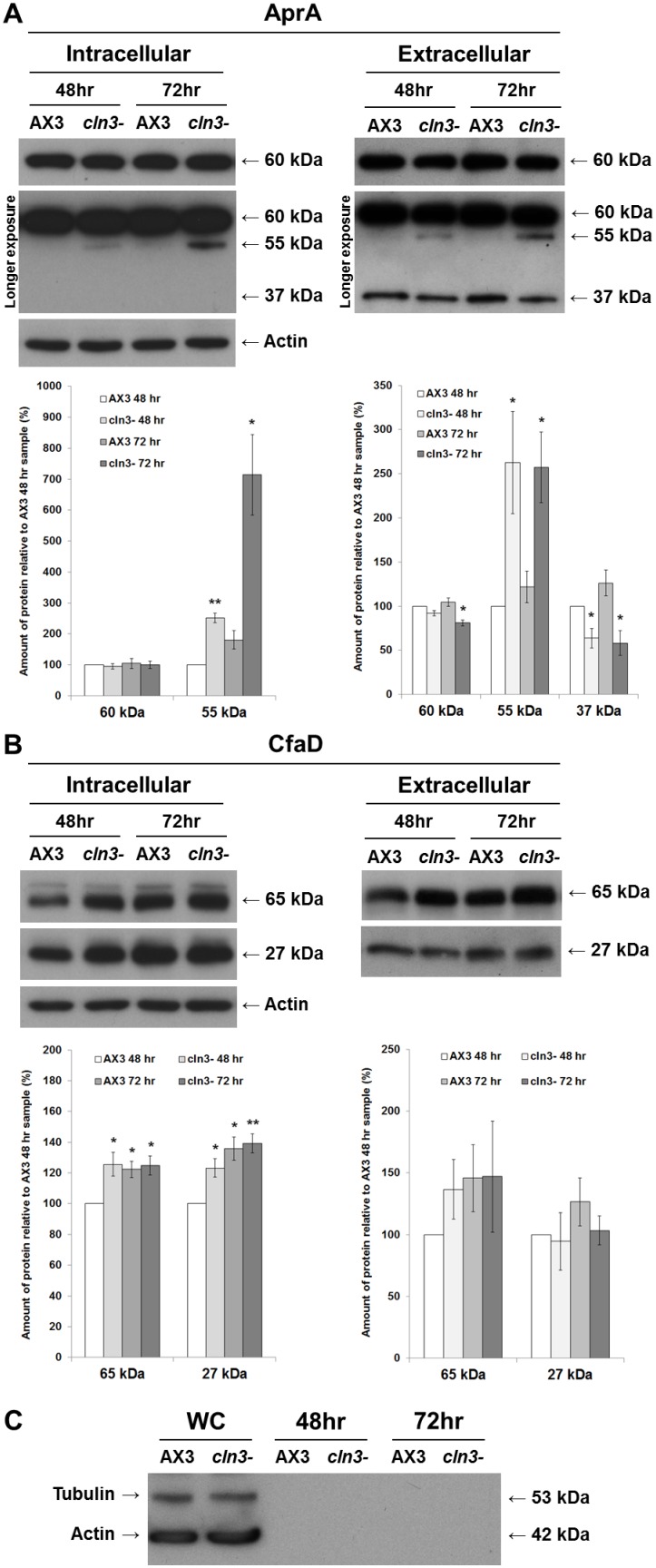
Effect of *cln3* knockout on the intra- and extracellular levels of AprA and CfaD. AX3 and *cln3^−^* cells grown axenically in HL5 were harvested and lysed after 48 and 72 hours of growth. Whole cell lysates (20 µg) (i.e., intracellular) and samples of conditioned growth media (i.e., extracellular) were separated by SDS-PAGE and analyzed by western blotting with anti-AprA, anti-CfaD, anti-tubulin, and anti-actin. Molecular weight markers (in kDa) are shown to the right of each blot. (A) Intra- and extracellular protein levels of AprA. Immunoblots that were exposed for a longer period of time (i.e., longer exposure) are included to show the 55-kDa and 37-kDa protein bands detected by anti-AprA. Note that the 37-kDa protein was detected in samples of conditioned growth media, but not in whole cell lysates. (B) Intra- and extracellular protein levels of CfaD. Data in all plots presented as mean amount of protein relative to AX3 48 hour sample (%) ± s.e.m (n = 4 independent experimental means, from 2 replicates in each experiment). Statistical significance was determined using a one-sample t-test (mean, 100; two-tailed) vs. the AX3 48 hour sample. *p-value<0.05. **p-value<0.01. (C) Detection of tubulin and actin in whole cell lysates (WC; lanes 1–2), but not in samples of conditioned growth media (lanes 3–6).

In an attempt to gain further insight into the possible mechanisms by which Cln3 deficiency leads to enhanced proliferation, we next investigated two secreted proteins that modulate growth in *Dictyostelium* by repressing cell proliferation: autocrine proliferation repressor A (AprA) and counting factor-associated protein D (CfaD) [41], [42]. Whole cell lysates (i.e., intracellular) and conditioned growth media (i.e., extracellular) from AX3 and *cln3^−^* cells were analyzed for the levels of AprA and CfaD present in each sample. In whole cell lysates, anti-AprA strongly detected a 60-kDa protein and weakly detected a 55-kDa protein (Fig. 6A), consistent with the banding pattern observed in another parental strain of *Dictyostelium*, AX2 [41]. After 48 and 72 hours of axenic growth, the amount of the 55-kDa protein in *cln3^−^* whole cell lysates was significantly greater than the amount in AX3 cells (Fig. 6A). In contrast, there were no significant differences in levels of the 60-kDa protein (Fig. 6A). In samples of conditioned growth media, anti-AprA detected the 60-kDa and 55 kDa proteins as well as a 37-kDa protein, which had not been observed in whole cell lysates from either AX3 or *cln3^−^* cells (Fig. 6A). After 72 hours of growth, the amount of 60-kDa protein in *cln3^−^* conditioned media, was significantly reduced compared to the amount present in AX3 conditioned media (Fig. 6A). After 48 and 72 hours of growth, the amount of 37-kDa protein in conditioned media from *cln3^−^* cells was also significantly reduced compared to amounts present in AX3 conditioned media (Fig. 6A). In contrast, the 55-kDa protein was present in significantly greater amounts at each time point in *cln3^−^* conditioned media (Fig. 6A).

In whole cell lysates and samples of conditioned growth media, anti-CfaD detected two proteins of molecular weights 65-kDa and 27-kDa, consistent with the predicted molecular weights of full-length CfaD and its putative cleavage product (Fig. 6B) [42]. After 48 hours of growth, there was significantly more CfaD (i.e., both 65-kDa and 27-kDa proteins) in *cln3^−^* whole cell lysates compared to AX3 lysates (Fig. 6B). However, there was no significant difference between strains in the intracellular level of either protein after 72 hours of growth (Fig. 6B). There was no significant effect resulting from Cln3 deficiency on the amounts of full-length CfaD or its cleavage product in conditioned media after 48 and 72 hours of growth (Fig. 6B). The absence of actin and tubulin from samples of conditioned growth media verified that the samples were not contaminated with intracellular proteins (Fig. 6C). Together, these data suggest that Cln3 deficiency in *Dictyostelium* leads to an enhanced rate of cell proliferation that is concomitant with alterations in secretory proteins that regulate extracellular proliferation signaling.

### Cln3 deficiency accelerates the formation of tipped mounds and slugs during mid-development

**Figure 7:**
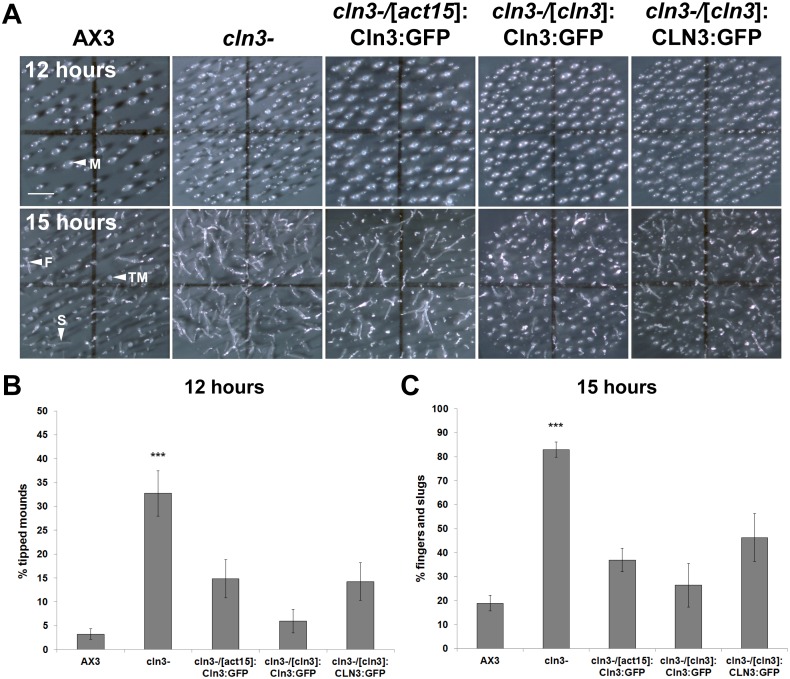
Effect of *cln3* knockout on the formation of tipped mounds and slugs. (A) AX3, *cln3^−^*, or *cln3^−^* cells overexpressing GFP-Cln3 or expressing GFP-Cln3 or GFP-CLN3 under the control of the *cln3* upstream element imaged after 12 and 15 hours of development. Images are a top-view of developing cells. (B) Quantification of the number of tipped mounds observed after 12 hours of development. Data presented as mean % tipped mounds ± s.e.m (n = 10–19). (C) Quantification of the number of fingers and slugs observed after 15 hours of development. Data presented as mean % fingers and slugs ± s.e.m (n = 10–33). Statistical significance was assessed using the Kruskal-Wallis test followed by the Dunn multiple comparison test (***p-value<0.001 vs. AX3). Scale bars = 1 mm. M, mound; TM, tipped-mound; F, finger; S, slug.

Given the dramatic increase in c*ln3* expression upon entering developmental phases of the *Dictyostelium* life cycle, we next sought to extend our analysis of Cln3 function to developmental processes. After 12 hours of development, 33±5% of *cln3^−^* structures had progressed to the tipped mound stage of development, compared to only 3±1% of AX3 structures (Fig. 7A,B). By 15 hours, 83±3% of *cln3^−^* multicellular structures had developed into either fingers or slugs compared to only 19±3% of AX3 structures (Fig. 7A, C). Overexpression of *Dictyostelium* GFP-Cln3, or expression of *Dictyostelium* GFP-Cln3 or human GFP-CLN3 under the control of the *cln3* upstream element in *cln3^−^* cells, suppressed the precocious development of *cln3^−^* cells at both the 12- and 15-hour time points to levels that were not significantly different from AX3 (Fig. 7A–C). Thus, Cln3 deficiency leads to precocious mid-stage development of *Dictyostelium* and this acceleration can be returned to near-normal levels by re-introducing *Dictyostelium* Cln3 or human CLN3 in an N-terminal fusion with GFP.

### Cln3 deficiency increases slug migration and accelerates fruiting body formation during late development

**Figure 8:**
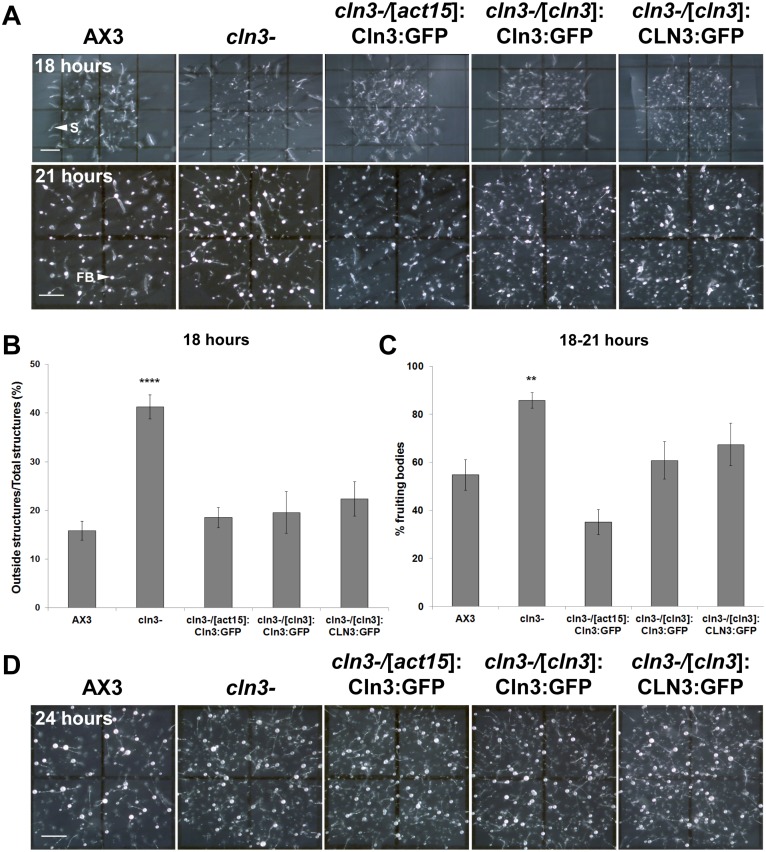
Effect of *cln3* knockout on slug migration and fruiting body formation. (A) AX3, *cln3^−^*, or *cln3^−^* cells overexpressing GFP-Cln3 or expressing GFP-Cln3 or GFP-CLN3 under control of the *cln3* upstream element imaged after 18 and 21 hours of development. (B) Quantification of the number of slugs that migrated outside the spot of deposition after 18 hours. Data presented as mean outside structures/total structures (%) ± s.e.m (n = 10–28). (C) Quantification of the number of fruiting bodies observed after 18–21 hours of development. Data presented as mean % fruiting bodies ± s.e.m (n = 10–32). (D) Fruiting bodies formed after 24 hours of development. Images in A and D are a top-view of developing cells. Statistical significance in B was assessed using one-way ANOVA (p<0.0001) followed by the Bonferroni multiple comparison test (****p-value<0.0001 vs. AX3). Statistical significance in C was assessed using the Kruskal-Wallis test followed by the Dunn multiple comparison test (**p-value<0.01 vs. AX3). Scale bars = 1 mm. S, slug; FB, fruiting body.

During the later stages of *Dictyostelium* development, a larger number of *cln3^−^* slugs were observed to migrate outside the spot of deposition compared to AX3 slugs (Fig. 8A). After 18 hours, 41±2% of *cln3^−^* slugs migrated out of the spot of deposition compared to only 16±2% of AX3 slugs (Fig. 8B). Notably, this could not be accounted for by the overall accelerated rate of development observed in *cln3^−^* cells, since a significantly higher percentage of *cln3^−^* slugs also migrated out of the spot after 21 hours compared to AX3 slugs (Fig. 8A, unpublished data). Overexpression of *Dictyostelium* GFP-Cln3, or expression of *Dictyostelium* GFP-Cln3 or human GFP-CLN3 under the control of the *cln3* upstream element in *cln3^−^* cells, significantly suppressed this slug migration phenotype to levels observed for AX3 slugs (Fig. 8A,B). Interestingly, the slug migration phenotype could not be explained by a defect in phototaxis, since we observed no obvious effect of *cln3* knockout on slug migration in a phototaxis assay (unpublished data).

Finally, Cln3 deficiency significantly accelerated fruiting body formation for those structures that remained in the deposition spot. After 18–21 hours of development, 86±3% of *cln3^−^* structures had developed into fruiting bodies compared to only 55±6% of AX3 structures (Fig. 8A, C). As it did for the slug migration stage, overexpression of *Dictyostelium* GFP-Cln3 or expression of *Dictyostelium* GFP-Cln3 or human GFP-CLN3 under the control of the *cln3* upstream element, in *cln3^−^* cells, significantly suppressed the accelerated fruiting body formation to levels that were not significantly different from AX3 (Fig. 8C).

Taken together, these data strongly indicate that Cln3 deficiency causes an overall accelerated rate of development in *Dictyostelium*, but that development nevertheless proceeds to the fruiting body stage (Fig. 8D). The ability to rescue the precocious development of *cln3^−^* cells by introducing human CLN3 strongly supports the notion that these steps require a function that is conserved between *Dictyostelium* and humans.

### Calcium chelation restores the timing of *cln3^−^* slug formation and suppresses the abnormal migration of *cln3^−^* slugs

**Figure 9:**
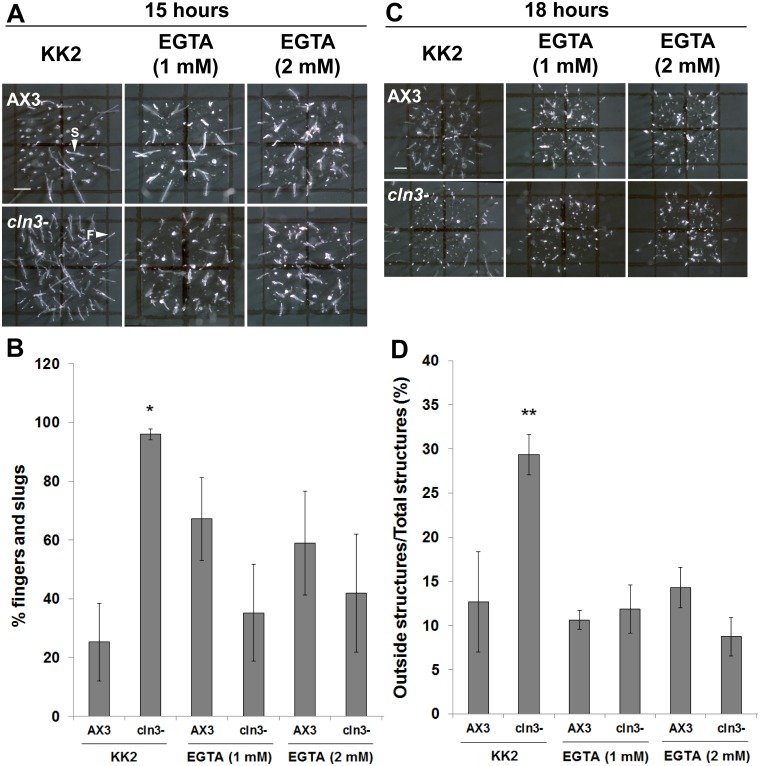
Effect of calcium chelation on AX3 and *cln3^−^* slug formation and migration. (A) AX3 and *cln3^−^* cells developed in the presence of KK2± EGTA and imaged after 15 hours of development. Scale bar = 1 mm. (B) Quantification of the number of fingers and slugs observed after 15 hours of development. Data presented as mean % fingers and slugs ± s.e.m (n≥4). (C) AX3 and *cln3^−^* cells developed in the presence of KK2± EGTA and imaged after 18 hours of development. Scale bar = 1 mm. (D) Quantification of the number of slugs that migrated outside the spot of deposition after 18 hours. Data presented as mean outside structures/total structures (%) ± s.e.m (n≥5). Images in A and C are a top-view of developing cells. Statistical significance in B was assessed using the Kruskal-Wallis test followed by the Dunn multiple comparison test (*p-value<0.05 vs. AX3). Statistical significance in D was assessed using one-way ANOVA (p<0.0001) followed by the Bonferroni multiple comparison test (**p-value<0.01 vs. AX3). F, finger; S, slug.

Since calcium signaling has been shown to be involved in regulating a number of developmental processes in *Dictyostelium* [49]–[52], the effect of calcium chelation on the substantial acceleration of mid-developmental events in *cln3^−^* cells was assessed. AX3 and *cln3^−^* cells were deposited on filters soaked in EGTA at concentrations that have previously been shown to be effective at chelating calcium during *Dictyostelium* development [51], [52]. The timing of slug formation and the extent of slug migration were then assessed. Interestingly, EGTA (1 mM and 2 mM) suppressed the accelerated formation of *cln3^−^* slugs and fingers after 15 hours of development, and suppressed the enhanced migration of *cln3^−^* slugs at the 18-hour time point to levels that were not significantly different from AX3 (Fig. 9A–D). EGTA had no significant effect on the accelerated formation of *cln3^−^* fruiting bodies (unpublished data).

## Discussion

In this study, we have shown that *Dictyostelium* contains an ortholog of CLN3, for which loss-of-function mutations in humans causes the childhood onset neurodegenerative disorder JNCL. We generated a *Dictyostelium cln3* knockout mutant that was validated by PCR and Southern blotting and have provided evidence that links Cln3 function to axenic growth and multicellular development. *Dictyostelium* GFP-Cln3 localizes primarily to the CV system, and to a lesser extent, to compartments of the endocytic pathway. Expression of *Dictyostelium* GFP-Cln3 or human GFP-CLN3 in *cln3^−^* cells suppresses the aberrant proliferation, precocious development, and slug migration phenotypes observed in knockout cells. Together, our data strongly suggest that Cln3 is a negative regulator of proliferation and development in *Dictyostelium*. Finally, we have provided evidence linking AprA secretion and cleavage to Cln3 function during growth, and calcium signaling to Cln3 function during multicellular development.

The enhanced proliferation of *cln3^−^* cells, coupled with the observation that *Dictyostelium* GFP-Cln3 overexpression in AX3 cells significantly reduces the final density of stationary phase cultures, strongly support the notion that Cln3 negatively regulates this cellular process in *Dictyostelium*. In *Dictyostelium*, extracellular liquid is ingested by macropinocytosis. [53]. An increased rate of pinocytosis would conceivably allow cells to ingest nutrients required for growth at an enhanced rate. Moreover, Journet et al. [54] identified Cln3 in an analysis of the macropinocytic proteome of *Dictyostelium* amoeba. Our pinocytosis analysis of *cln3^−^* cells during axenic growth only revealed minor differences suggesting further work is needed to fully elucidate the mechanisms by which Cln3 deficiency affects cell proliferation in *Dictyostelium.* In other systems, CLN3 has also been reported to localize to the endocytic pathway and its deficiency impairs endocytosis in those systems [19], [55]–[59]. Together, our results, coupled with those reported by others, indicate that further research is required to determine the precise function of CLN3 in the endocytic pathway, which may be organism or cell-type dependent.

Based on our observations of the intra- and extracellular amounts of AprA and the fact that AprA negatively regulates cell proliferation in *Dictyostelium* [41], it would appear that the enhanced proliferation of *cln3^−^* cells could be at least partially explained by the lack of full-length AprA and its putative 37-kDa cleavage product in conditioned media. Since the intracellular amounts of 60-kDa AprA were not significantly different between AX3 and *cln3^−^* cells, thus excluding the possibility that *aprA* gene expression or translation were affected by Cln3 deficiency, our results suggest that Cln3 facilitates the secretion of AprA during growth. The detection of a 37-kDa protein by the highly specific anti-AprA antibody in conditioned media, but not whole cell lysates, suggests that AprA is cleaved extracellularly during growth. Since the amount of the 37-kDa protein was significantly reduced in *cln3^−^* cells, these results suggest that Cln3 deficiency also negatively affects the secretion of a protease required for AprA cleavage. This is supported by previous studies that have reported the proteolytic cleavage of extracellular proteins during growth and development [60]–[63]. Furthermore, a study describing the secreted proteome profile of growing and developing *Dictyostelium* cells also reports the detection of a large number of extracellular proteases in conditioned media [64]. Like AprA, CfaD is part of a ∼150 kDa complex that functions extracellularly to repress cell proliferation in *Dictyostelium,* and chromatography and pull-down assays suggest that CfaD interacts with AprA [42]. Since increased levels of intracellular CfaD were observed in *cln3^−^* cells during the early stages of axenic growth, our results suggest that altered CfaD secretion could also explain the enhanced proliferation of *cln3^−^* cells. However, we observed no correlated decrease in the extracellular levels of CfaD over the same time period. Nevertheless, our data indicate that Cln3 facilitates the secretion of AprA, and may to a lesser extent, also facilitate CfaD secretion. Taken together, the altered secretion of these extracellular signaling proteins could explain the enhanced proliferation of *cln3^−^* cells.

During growth, *Dictyostelium* GFP-Cln3 localized primarily to the CV system in live and fixed cells, and to a lesser extent to the endocytic system. In *Dictyostelium*, the CV system is dynamic and functions in a number of cellular processes including osmoregulation, calcium storage, protein transport to the plasma membrane, and secretion [45], [65], [66]. Although *Dictyostelium* GFP-Cln3 was observed to localize to the CV system, we observed no obvious sensitivity of *cln3^−^* cells to hypo-osmotic conditions during growth in HL5 (25% HL5, 75% double-distilled water) or during starvation in double-distilled water (unpublished data). However, further analysis is required to determine if there are subtle effects of Cln3-deficiency on osmoregulation during *Dictyostelium* growth. In *Dictyostelium*, the CV and endosomal systems appear to be physically separated from each other. However, some experimental evidence also indicates that controlled intracellular transport might occur between these two systems [67]–[69]. The observation that GFP-Cln3 localizes to both the CV and endocytic systems in *Dictyostelium* is consistent with the localization of mammalian CLN3 to multiple subcellular compartments including the endocytic and lysosomal systems [13]. Notably, endogenous Cln3 has been reported within fractions of the macropinocytic pathway in *Dictyostelium*, consistent with our localization data presented here [3], [46], [47], [53]. Finally, since *Dictyostelium* GFP-Cln3 is able to rescue growth and developmental phenotypes, we are confident that we have correctly identified the subcellular localization of Cln3 in *Dictyostelium*.

Phenotypes were observed in *cln3^−^* cells during mid- and late *Dictyostelium* development that further support Cln3 as a negative regulator in *Dictyostelium*. Consistent with the relatively higher expression of *cln3* mRNA during mid- and late development, loss of *cln3* by gene knockout significantly accelerated the formation of mid- and late developmental structures. Precocious development has been observed in a number of *Dictyostelium* knockout mutants. Specifically, early tipped mound formation has been reported in strains overexpressing cyclin C, cyclin-dependent kinase 8, or the G-protein alpha 5 subunit, and in knockout mutants of histidine kinase C, a metabotropic glutamate receptor-like protein, protein inhibitor of STAT, and SCAR/WAVE [70]–[75]. Several knockout mutants that display increased slug migration have been described, including mutants for genes important for oxysterol binding, the assembly of mitochondrial complex I, and the targeting of proteins for degradation via proteasomes [76]–[78]. This phenotype has also been observed in cells overexpressing histidine kinase C or in cells where calcium-binding protein 3 expression has been knocked down by RNAi [72], [79]. The diversity of functions associated with these proteins as well as those discussed above for the other developmental phenotypes in *cln3^−^* cells, highlight the importance of elucidating the signal transduction pathways underlying the function of Cln3 during *Dictyostelium* development.

The ability to completely restore the timing of *cln3^−^* slug formation and the enhanced slug migration through the chelation of calcium provides some mechanistic insight into the signaling pathways affected by Cln3 deficiency during these stages of the life cycle. These results are interesting given that *Dictyostelium* GFP-Cln3 localizes predominantly to the CV system, which has been shown to be a highly efficient store of intracellular calcium, and to be required for cAMP-induced calcium influx [65]. In addition, the primary sensor of intracellular calcium, calmodulin, is found predominantly on the membranes of the CV system [80], [81]. Our results are consistent with studies in mammalian systems that have reported altered calcium homeostasis in the absence of functional CLN3, which may lead to synaptic dysfunction and neuronal apoptosis [82]–[83]. Furthermore, CLN3 has been shown to bind to the neuronal calcium-binding protein, calsenilin, in a calcium-dependent manner [84].

Taken together, our data strongly supports Cln3 as a negative regulator of proliferation and development in *Dictyostelium*. Furthermore, our study indicates that *cln3* knockout in *Dictyostelium* compromises the cell’s ability to respond to extracellular and/or environmental cues. This first report of a *Dictyostelium* model to study NCL should spur further research using this important model organism. In addition to *CLN3*, *Dictyostelium* also possesses homologs to most of the other known NCL genes (e.g., *CLN1-5, CLN7, CLN10-14*) indicating that the NCL biological pathway is likely to be conserved in this model system. The cellular processes and signaling pathways that regulate the behavior of *Dictyostelium* cells are remarkably similar to those observed in human cells, strengthening the argument that investigation of NCL gene function in this model organism offers something unique to the study of this devastating group of inherited neurodegenerative disorders.

## Supporting Information

Figure S1 **Analysis of gene expression driven by endogenous** ***cln3*** **upstream elements.** AX3 cells were transformed with the appropriate construct (pTX-GFP; *act15* promoter replaced with *cln3* upstream element 1, 2, or 3) and grown in HL5. Cells were harvested and lysed. Proteins (20 µg) were separated by SDS-PAGE and analyzed by western blotting with anti-GFP, anti-tubulin (loading control), or anti-actin (loading control). Molecular weight markers (in kDa) are shown to the right of each blot.
(TIF)

Figure S2 **Western blot analysis of** ***Dictyostelium*** **strains expressing** ***Dictyostelium*** **GFP-Cln3 or human GFP-CLN3 under the control of the** ***act15*** **promoter or** ***cln3*** **upstream element 1.** (A–C) AX3 and *cln3^−^* cells were transformed with the appropriate construct (gene expression driven by the *act15* promoter) and grown in HL5. Cells were lysed and sample loading buffer was added to whole cell lysates which were either loaded directly into polyacrylamide gels or heated for 5 minutes at 95°C prior to loading into gels. Proteins (20 µg) were separated by SDS-PAGE and analyzed by western blotting with anti-GFP, anti-tubulin (loading control), or anti-actin (loading control). (D) AX3 and *cln3^−^* cells were transformed with the appropriate construct (gene expression driven by *cln3* upstream element 1) and grown in HL5. Cells were lysed and samples were prepared and analyzed as described above. Molecular weight markers (in kDa) are shown to the left of each blot.
(TIF)

Figure S3 **Video of** ***Dictyostelium*** **GFP-Cln3 localization in AX3 cells incubated in water.** AX3 cells expressing *Dictyostelium* GFP-Cln3 were grown overnight in low-fluorescence HL5. Cells were washed two times with double distilled water and then resuspended in double distilled water.
(MPG)

Table S1 **List of primers used for** ***cln3*** **knockout validation and amplification of** ***cln3*** **upstream elements.** The following primers were designed to amplify gDNA from AX3 and *cln3^−^* cells to validate the knockout of the *cln3* gene in the *bsr* resistant clone and to amplify fragments upstream of the *cln3* start site. The *Dictyostelium* gene denoted DDB_G0291155 lies downstream of *cln3* and was amplified to confirm that the insertion of the *bsr* cassette did not affect gene DDB_G0291155.
(DOCX)

File S1 **Results, Discussion, and References specific to the Supplemental Table and Figures.**
(DOCX)

## References

[1] Santavuori P (1988) Neuronal ceroid lipofuscinosis in childhood. Brain Dev 10: 80–83. 3291628 10.1016/s0387-7604(88)80075-5

[2] Anderson GW, Goebel HH, Simonati A (2013) Human pathology in NCL. Biochim Biophys Acta 1832: 1807–1826. 23200925 10.1016/j.bbadis.2012.11.014

[3] Kollmann K, Uusi-Rauva K, Scifo E, Tyynelä J, Jalanko A, et al (2013) Cell biology and function of neuronal ceroid lipofuscinosis-related proteins. Biochim Biophys Acta 1832: 1866–1881. 23402926 10.1016/j.bbadis.2013.01.019

[4] Schulz A, Kohlschütter A, Mink J, Simonati A, Williams R (2013) NCL diseases - clinical perspectives. Biochim Biophys Acta 1832: 1801–1806. 23602993 10.1016/j.bbadis.2013.04.008 PMC4631127

[5] Hofman I, van der Wal A, Dingemans K, Becker A (2001) Cardiac pathology in neuronal ceroid lipofuscinoses – a clinicopathologic correlation in three patients. Eur J Paediatr Neurol 5(Suppl A): 213–217. 10.1053/ejpn.2000.0465 11589001

[6] Ostergaard J, Rasmussen T, Molgaard H (2011) Cardiac involvement in juvenile neuronal ceroid lipofuscinosis (Batten disease). Neurology 76: 1245–1251. 21464428 10.1212/WNL.0b013e31821435bd

[7] Chattopadhyay S, Ito M, Cooper JD, Brooks AI, Curran TM, et al (2002) An autoantibody inhibitory to glutamic acid decarboxylase in the neurodegenerative disorder Batten disease. Hum Mol Genet 11: 1421–1431. 12023984 10.1093/hmg/11.12.1421

[8] Castaneda J, Pearce D (2008) Identification of alpha-fetoprotein as an autoantigen in juvenile Batten disease. Neurobiol Dis 29: 92–102. 17931875 10.1016/j.nbd.2007.08.007

[9] Haltia M, Goebel HH (2013) The neuronal ceroid-lipofuscinoses: A historical introduction. Biochim Biophys Acta 1832: 1795–1800. 22959893 10.1016/j.bbadis.2012.08.012

[10] The International Batten Disease Consortium (1995) Isolation of a novel gene underlying Batten disease, *CLN3* . Cell 82: 949–957. 7553855 10.1016/0092-8674(95)90274-0

[11] Mole S (2012) NCL Mutation and Patient Database. NCL Resource – A Gateway for Batten Disease. Available: http://www.ucl.ac.uk/ncl/mutation.shtml.

[12] Warrier V, Vieira M, Mole SE (2013) Genetic basis and phenotypic correlations of the neuronal ceroid lipofusinoses. Biochim Biophys Acta 1832: 1827–1830. 23542453 10.1016/j.bbadis.2013.03.017

[13] Cotman SL, Staropoli JF (2012) The juvenile Batten disease protein, CLN3, and its role in regulating anterograde and retrograde post-Golgi trafficking. Clin Lipidol 7: 79–91. 22545070 10.2217/clp.11.70 PMC3334816

[14] Uusi-Rauva K, Kyttälä A, van der Kant R, Vesa J, Tanhuanpää K, et al (2012) Neuronal ceroid lipofuscinosis protein CLN3 interacts with motor proteins and modifies location of late endosomal compartments. Cell Mol Life Sci 69: 2075–2089. 22261744 10.1007/s00018-011-0913-1 PMC11114557

[15] Luiro K, Kopra O, Lehtovirta M, Jalanko A (2001) CLN3 protein is targeted to neuronal synapses but excluded from synaptic vesicles: new clues to Batten disease. Hum Mol Genet 10: 2123–2131. 11590129 10.1093/hmg/10.19.2123

[16] Pearce DA, Nosel SA, Sherman F (1999) Studies of pH regulation by Btn1p, the yeast homolog of human Cln3p. Mol Genet Metab 66: 320–323. 10191121 10.1006/mgme.1999.2819

[17] Gachet Y, Codlin S, Hyams JS, Mole SE (2005) btn1, the *Schizosaccharomyces pombe* homologue of the human Batten disease gene CLN3, regulates vacuole homeostasis. J Cell Sci 118: 5525–5536. 16291725 10.1242/jcs.02656

[18] Holopainen JM, Saarikoski J, Kinnunen PK, Järvelä I (2001) Elevated lysosomal pH in neuronal ceroid lipofuscinoses (NCLs). Eur J Biochem 268: 5851–5856. 11722572 10.1046/j.0014-2956.2001.02530.x

[19] Luiro K, Yliannala K, Ahtiainen L, Maunu H, Järvelä I, et al (2004) Interconnections of CLN3, Hook1 and Rab proteins link Batten disease to defects in the endocytic pathway. Hum Mol Genet 13: 3017–3027. 15471887 10.1093/hmg/ddh321

[20] Cao Y, Espinola JA, Fossale E, Massey AC, Cuervo AM, et al (2006) Autophagy is disrupted in a knock-in mouse model of juvenile neuronal ceroid lipofuscinosis. J Biol Chem 281: 20483–20493. 16714284 10.1074/jbc.M602180200

[21] Getty AL, Pearce DA (2011) Interactions of the proteins of neuronal ceroid lipofuscinosis: clues to function. Cell Mol Life Sci 68: 453–474. 20680390 10.1007/s00018-010-0468-6 PMC4120758

[22] van Egmond WN, Kortholt A, Plak K, Bosgraaf L, Bosgraaf S, et al (2008) Intramolecular activation mechanism of the *Dictyostelium* LRRK2 homolog Roco protein GbpC. J Biol Chem 283: 30412–30420. 18703517 10.1074/jbc.M804265200 PMC2662088

[23] Gilsbach BK, Ho FY, Vetter IR, van Haastert PJ, Wittinghofer A, et al (2012) Roco kinase structures give insights into the mechanism of Parkinson disease-related leucine-rich-repeat kinase 2 mutations. Proc Natl Acad Sci USA 109: 10322–10327. 22689969 10.1073/pnas.1203223109 PMC3387044

[24] Meyer I, Kuhnert O, Gräf R (2011) Functional analyses of lissencephaly-related proteins in *Dictyostelium* . Semin Cell Dev Biol 22: 89–96. 21034843 10.1016/j.semcdb.2010.10.007

[25] McMains VC, Myre M, Kreppel L, Kimmel AR (2010) *Dictyostelium* possesses highly diverged presenilin/gamma-secretase that regulates growth and cell-fate specification and can accurately process human APP: a system for functional studies of the presenilin/gamma-secretase complex. Dis Model Mech 3: 581–594. 20699477 10.1242/dmm.004457 PMC2931536

[26] Myre MA, Lumsden AL, Thompson MN, Wasco W, MacDonald ME, et al (2011) Deficiency of huntingtin has pleiotropic effects in the social amoeba *Dictyostelium discoideum* . PLoS Genet 7: e1002052. 21552328 10.1371/journal.pgen.1002052 PMC3084204

[27] Lo Sardo V, Zuccato C, Gaudenzi G, Vitali B, Ramos C, et al (2012) An evolutionary recent neuroepithelial cell adhesion function of huntingtin implicates ADAM10-Ncadherin. Nat Neurosci 15: 713–721. 22466506 10.1038/nn.3080

[28] Myre MA (2012) Clues to γ-secretase, huntingtin and Hirano body normal function using the model organism *Dictyostelium discoideum* . J Biomed Sci 10: 19–41. 10.1186/1423-0127-19-41 PMC3352040 22489754

[29] Maniak M (2011) *Dictyostelium* as a model for human lysosomal and trafficking diseases. *Semin.* . Cell Dev Biol 22: 114–119. 10.1016/j.semcdb.2010.11.001 21056680

[30] Huber RJ, O’Day DH (2012) A matricellular protein and EGF-like repeat signalling in the social amoebozoan *Dictyostelium discoideum* . Cell Mol Life Sci 69: 3989–3997. 22782112 10.1007/s00018-012-1068-4 PMC11115030

[31] Muller-Taubenberger A, Kortholt A, Eichinger L (2013) Simple system - substantial share: The use of *Dictyostelium* in cell biology and molecular medicine. Eur J Cell Biol 92: 45–53. 23200106 10.1016/j.ejcb.2012.10.003

[32] Huber RJ (2014) The cyclin-dependent kinase family in the social amoebozoan *Dictyostelium discoideum* . Cell Mol Life Sci 71: 629–639. 23974243 10.1007/s00018-013-1449-3 PMC11113532

[33] Levi S, Polyakov M, Egelhoff TT (2000) Green fluorescent protein and epitope tag fusion vectors for *Dictyostelium discoideum* . Plasmid 44: 231–238. 11078649 10.1006/plas.2000.1487

[34] Rivero F, Maniak M (2006) Quantitative and microscopic methods for studying the endocytic pathway. In: Eichinger L, Rivero F, editors. Methods in Molecular Biology 346: Dictyostelium discoideum Protocols. New Jersey: Humana Press/Totowa. 423–438. 10.1385/1-59745-144-4:423 16957305

[35] Huber RJ, O'Day DH (2012) The cyclin-dependent kinase inhibitor roscovitine inhibits kinase activity, cell proliferation, multicellular development, and Cdk5 nuclear translocation in *Dictyostelium discoideum* . J Cell Biochem 113: 868–876. 22234985 10.1002/jcb.23417

[36] Huber RJ, O'Day DH (2011) Nucleocytoplasmic transfer of cyclin dependent kinase 5 and its binding to puromycin-sensitive aminopeptidase in *Dictyostelium discoideum* . Histochem Cell Biol 136: 177–189. 21766205 10.1007/s00418-011-0839-6

[37] Charette SJ, Cosson P (2006) Exocytosis of late endosomes does not directly contribute membrane to the formation of phagocytic cups or pseudopods in *Dictyostelium* . FEBS Lett 580: 4923–4928. 16920105 10.1016/j.febslet.2006.08.009

[38] Fok AK, Clarke M, Ma L, Allen RD (1993) Vacuolar H+-ATPase of *Dictyostelium discoideum*. A monoclonal antibody study. J Cell Sci 106: 1103–1113. 8126094 10.1242/jcs.106.4.1103

[39] Benghezal M, Gotthardt D, Cornillon S, Cosson P (2001) Localization of the Rh50-like protein to the contractile vacuole in *Dictyostelium* . Immunogenetics 52: 284–288. 11220631 10.1007/s002510000279

[40] Ravanel K, de Chassey B, Cornillon S, Benghezal M, Zulianello L, et al (2001) Membrane sorting in the endocytic and phagocytic pathway of *Dictyostelium discoideum* . Eur J Cell Biol 80: 754–764. 11831389 10.1078/0171-9335-00215

[41] Brock DA, Gomer RH (2005) A secreted factor represses cell proliferation in *Dictyostelium* . Development 132: 4553–4562. 16176950 10.1242/dev.02032 PMC4484793

[42] Bakthavatsalam D, Brock DA, Nikravan NN, Houston KD, Hatton RD, et al (2008) The secreted *Dictyostelium* protein CfaD is a chalone. J Cell Sci 121: 2473–2480. 18611962 10.1242/jcs.026682 PMC2716657

[43] Faix J, Kreppel L, Shaulsky G, Schleicher M, Kimmel AR (2004) A rapid and efficient method to generate multiple gene disruptions in *Dictyostelium discoideum* using a single selectable marker and the Cre-loxP system. Nucleic Acids Res 32: e143. 15507682 10.1093/nar/gnh136 PMC528815

[44] Haskell RE, Derksen TA, Davidson BL (1999) Intracellular trafficking of the JNCL protein CLN3. Mol Genet Metab 66: 253–260. 10191111 10.1006/mgme.1999.2802

[45] Gerisch G, Heuser J, Clarke M (2002) Tubular-vesicular transformation in the contractile vacuole system of *Dictyostelium* . Cell Biol Int 26: 845–852. 12421575 10.1006/cbir.2002.0938

[46] Rodriguez-Paris JM, Nolta KV, Steck TL (1993) Characterization of lysosomes isolated from *Dictyostelium discoideum* by magnetic fractionation. J Biol Chem 268: 9110–9116. 7682559

[47] Temesvari L, Rodriguez-Paris J, Bush J, Steck TL, Cardelli J (1994) Characterization of lysosomal membrane proteins of *Dictyostelium discoideum*. A complex population of acidic integral membrane glycoproteins, Rab GTP-binding proteins and vacuolar ATPase subunits. J Biol Chem 269: 25719–25727. 7929276

[48] Rot G, Parikh A, Curk T, Kuspa A, Shaulsky G, et al (2009) dictyExpress: A *Dictyostelium discoideum* gene expression database with an explorative data analysis web-based interface. BMC Bioinformatics 10: 256. 19706156 10.1186/1471-2105-10-265 PMC2738683

[49] Sakamoto H, Nishio K, Tomisako M, Kuwayama H, Tanaka Y, et al (2003) Identification and characterization of novel calcium-binding proteins of *Dictyostelium* and their spatial expression patterns during development. Dev Growth Differ 45: 507–514. 14706075 10.1111/j.1440-169x.2003.00718.x

[50] Scherer A, Kuhl S, Wessels D, Lusche DF, Raisley B, et al (2010) Ca^2+^ chemotaxis in *Dictyostelium discoideum* . J Cell Sci 123: 3756–3767. 20940253 10.1242/jcs.068619

[51] Poloz Y, O'Day DH (2012) Ca^2+^ signaling regulates ecmB expression, cell differentiation and slug regeneration in *Dictyostelium* . Differentiation 84: 163–175. 22595345 10.1016/j.diff.2012.02.009

[52] Poloz Y, O'Day DH (2012) Colchicine affects cell motility, pattern formation and stalk cell differentiation in *Dictyostelium* by altering calcium signaling. Differentiation 83: 185–199. 22381626 10.1016/j.diff.2011.12.006

[53] Maniak M (2003) Fusion and fission events in the endocytic pathway of *Dictyostelium* . Traffic 4: 1–5. 12535269 10.1034/j.1600-0854.2003.40101.x

[54] Journet A, Klein G, Brugière S, Vandenbrouck Y, Chapel A, et al (2012) Investigating the macropinocytic proteome of *Dictyostelium* amoebae by high-resolution mass spectrometry. Proteomics 12: 241–245. 22120990 10.1002/pmic.201100313

[55] Fossale E, Wolf P, Espinola JA, Lubicz-Nawrocka T, Teed AM, et al (2004) Membrane trafficking and mitochondrial abnormalities precede subunit c deposition in a cerebellar cell model of juvenile neuronal ceroid lipofuscinosis. BMC Neurosci 5: 57. 15588329 10.1186/1471-2202-5-57 PMC539297

[56] Codlin S, Haines RL, Mole SE (2008) btn1 affects endocytosis, polarization of sterol-rich membrane domains and polarized growth in *Schizosaccharomyces pombe* . Traffic 9: 936–950. 18346214 10.1111/j.1600-0854.2008.00735.x PMC2440566

[57] Cao Y, Staropoli JF, Biswas S, Espinola JA, MacDonald ME, et al (2011) Distinct early molecular responses to mutations causing vLINCL and JNCL presage ATP synthase subunit C accumulation in cerebellar cells. PLoS One 6: e17118. 21359198 10.1371/journal.pone.0017118 PMC3040763

[58] Tecedor L, Stein CS, Schultz ML, Farwanah H, Sandhoff K, et al (2013) CLN3 loss disturbs membrane microdomain properties and protein transport in brain endothelial cells. J Neurosci 33: 18065–18079. 24227717 10.1523/JNEUROSCI.0498-13.2013 PMC3828460

[59] Vidal-Donet JM, Cárcel-Trullols J, Casanova B, Aguado C, Knecht E (2013) Alterations in ROS activity and lysosomal pH account for distinct patterns of macroautophagy in LINCL and JNCL fibroblasts. PLoS One 8: e55526. 23408996 10.1371/journal.pone.0055526 PMC3567113

[60] Suarez A, Huber RJ, Myre MA, O’Day DH (2011) An extracellular matrix, calmodulin-binding protein from *Dictyostelium* with EGF-like repeats that enhance cell motility. Cell Signal 23: 1197–1206. 21402150 10.1016/j.cellsig.2011.03.008

[61] Huber RJ, Suarez A, O'Day DH (2012) CyrA, a matricellular protein that modulates cell motility in *Dictyostelium discoideum* . Matrix Biol 31: 271–280. 22391412 10.1016/j.matbio.2012.02.003

[62] Brock DA, Gomer RH (1999) A cell-counting factor regulating structure size in *Dictyostelium* . Genes Dev 13: 1960–1969. 10444594 10.1101/gad.13.15.1960 PMC316923

[63] Brock DA, Hatton RD, Giurgiutiu DV, Scott B, Ammann R, et al (2002) The different components of a multisubunit cell number-counting factor have both unique and overlapping functions. Development 129: 3657–3668. 12117815 10.1242/dev.129.15.3657

[64] Bakthavatsalam D, Gomer RH (2010) The secreted proteome profile of developing *Dictyostelium discoideum* cells. Proteomics 10: 2556–2559. 20422638 10.1002/pmic.200900516 PMC2926150

[65] Malchow D, Lusche DF, Schlatterer C, De Lozanne A, Müller-Taubenberger A (2006) The contractile vacuole in Ca^2+^-regulation in *Dictyostelium*: its essential function for cAMP-induced Ca^2+^-influx. BMC Dev Biol 6: 31. 16787542 10.1186/1471-213X-6-31 PMC1513554

[66] Sriskanthadevan S, Lee T, Lin Z, Yang D, Siu CH (2009) Cell adhesion molecule DdCAD-1 is imported into contractile vacuoles by membrane invagination in a Ca^2+^- and conformation-dependent manner. J Biol Chem 284: 36377–36386. 19875452 10.1074/jbc.M109.057257 PMC2794753

[67] Hacker U, Albrecht R, Maniak M (1997) Fluid-phase uptake by macropinocytosis in *Dictyostelium* . J Cell Sci 110: 105–112. 9044041 10.1242/jcs.110.2.105

[68] Gabriel D, Hacker U, Köhler J, Müller-Taubenberger A, Schwartz JM, et al (1999) The contractile vacuole network of *Dictyostelium* as a distinct organelle: its dynamics visualized by a GFP marker protein. J Cell Sci 112: 3995–4005. 10547360 10.1242/jcs.112.22.3995

[69] Mercanti V, Charette SJ, Bennett N, Ryckewaert JJ, Letourneur F, et al (2006) Selective membrane exclusion in phagocytic and macropinocytic cups. J Cell Sci 119: 4079–4087. 16968738 10.1242/jcs.03190

[70] Greene DM, Hsu DW, Pears CJ (2010) Control of cyclin C levels during development of *Dictyostelium* . PLoS One 5: e10543. 20479885 10.1371/journal.pone.0010543 PMC2866538

[71] Hadwiger JA, Natarajan K, Firtel RA (1996) Mutations in the *Dictyostelium* heterotrimeric G protein alpha subunit G alpha5 alter the kinetics of tip morphogenesis. Development 122: 1215–1224. 8620848 10.1242/dev.122.4.1215

[72] Singleton CK, Zinda MJ, Mykytka B, Yang P (1998) The histidine kinase dhkC regulates the choice between migrating slugs and terminal differentiation in *Dictyostelium discoideum* . Dev Biol 203: 345–357. 9808785 10.1006/dbio.1998.9049

[73] Prabhu Y, Müller R, Anjard C, Noegel AA (2007) GrlJ, a *Dictyostelium* GABAB-like receptor with roles in post-aggregation development. BMC Dev Biol 7: 44. 17501984 10.1186/1471-213X-7-44 PMC1885808

[74] Kawata T, Hirano T, Ogasawara S, Aoshima R, Yachi A (2011) Evidence for a functional link between Dd-STATa and Dd-PIAS, a *Dictyostelium* PIAS homologue. Dev Growth Differ 53: 897–909. 21933174 10.1111/j.1440-169X.2011.01296.x

[75] Bear JE, Rawls JF, Saxe III CL (1998) SCAR, a WASP-related protein, isolated as a suppressor of receptor defects in late *Dictyostelium* development. J Cell Biol 142: 1325–1335. 9732292 10.1083/jcb.142.5.1325 PMC2149354

[76] Fukuzawa M, Williams JG (2002) OSBPa, a predicted oxysterol binding protein of *Dictyostelium*, is required for regulated entry into culmination. FEBS Lett 527: 37–42. 12220630 10.1016/s0014-5793(02)03150-2

[77] Torija P, Vicente JJ, Rodrigues TB, Robles A, Cerdán S, et al (2006) Functional genomics in *Dictyostelium*: MidA, a new conserved protein, is required for mitochondrial function and development. J Cell Sci 119: 1154–1164. 16507593 10.1242/jcs.02819

[78] Nelson MK, Clark A, Abe T, Nomura A, Yadava N, et al (2000) An F-Box/WD40 repeat-containing protein important for *Dictyostelium* cell-type proportioning, slug behaviour, and culmination. Dev Biol 224: 42–59. 10898960 10.1006/dbio.2000.9793

[79] Lee CH, Jeong SY, Kim BJ, Choi CH, Kim JS, et al (2005) *Dictyostelium* CBP3 associates with actin cytoskeleton and is related to slug migration. Biochim Biophys Acta 1743: 281–290. 15843041 10.1016/j.bbamcr.2005.01.003

[80] Zhu Q, Clarke M (1992) Association of calmodulin and an unconventional myosin with the contractile vacuole complex of *Dictyostelium discoideum* . J Cell Biol 118: 347–358. 1629238 10.1083/jcb.118.2.347 PMC2290049

[81] Sriskanthadevan S, Brar SK, Manoharan K, Siu CH (2013) Ca(^2+^) -calmodulin interacts with DdCAD-1 and promotes DdCAD-1 transport by contractile vacuoles in *Dictyostelium* cells. FEBS J 280: 1795–1806. 23441816 10.1111/febs.12203

[82] An Haack K, Narayan SB, Li H, Warnock A, Tan L, et al (2011) Screening for calcium channel modulators in CLN3 siRNA knock down SH-SY5Y neuroblastoma cells reveals a significant decrease of intracellular calcium levels by selected L-type calcium channel blockers. Biochim Biophys Acta 1810: 186–191. 20933060 10.1016/j.bbagen.2010.09.004 PMC3109357

[83] Warnock A, Tan L, Li C, An Haack K, Narayan SB, et al (2013) Amlodipine prevents apoptotic cell death by correction of elevated intracellular calcium in a primary neuronal model of Batten disease (CLN3 disease). Biochem Biophys Res Commun 436: 645–649. 23769828 10.1016/j.bbrc.2013.04.113

[84] Chang JW, Choi H, Kim HJ, Jo DG, Jeon YJ, et al (2007) Neuronal vulnerability of CLN3 deletion to calcium-induced cytotoxicity is mediated by calsenilin. Hum Mol Genet 16: 317–326. 17189291 10.1093/hmg/ddl466

[85] Bause E (1983) Structural requirements of N-glycosylation of proteins. Biochem J 209: 331–336. 6847620 10.1042/bj2090331 PMC1154098

[86] Munroe PB, Mitchison HM, O'Rawe AM, Anderson JW, Boustany RM, et al (1997) Spectrum of mutations in the Batten disease gene, *CLN3* . Am J Hum Genet 61: 310–316. 9311735 10.1086/514846 PMC1715900

[87] Haskell RE, Carr CJ, Pearce DA, Bennett MJ, Davidson BL (2000) Batten disease: evaluation of CLN3 mutations on protein localization and function. Hum Mol Genet 9: 735–744. 10749980 10.1093/hmg/9.5.735

