# Supplementary figures and images for: Loss of Cln3 Function in the Social Amoeba Dictyostelium discoideum Causes Pleiotropic Effects That Are Rescued by Human CLN3
Source: PLoS One. 2014 Oct 17;9(10):e110544. doi: 10.1371/journal.pone.0110544 (PMC4201555; doi:10.1371/journal.pone.0110544)

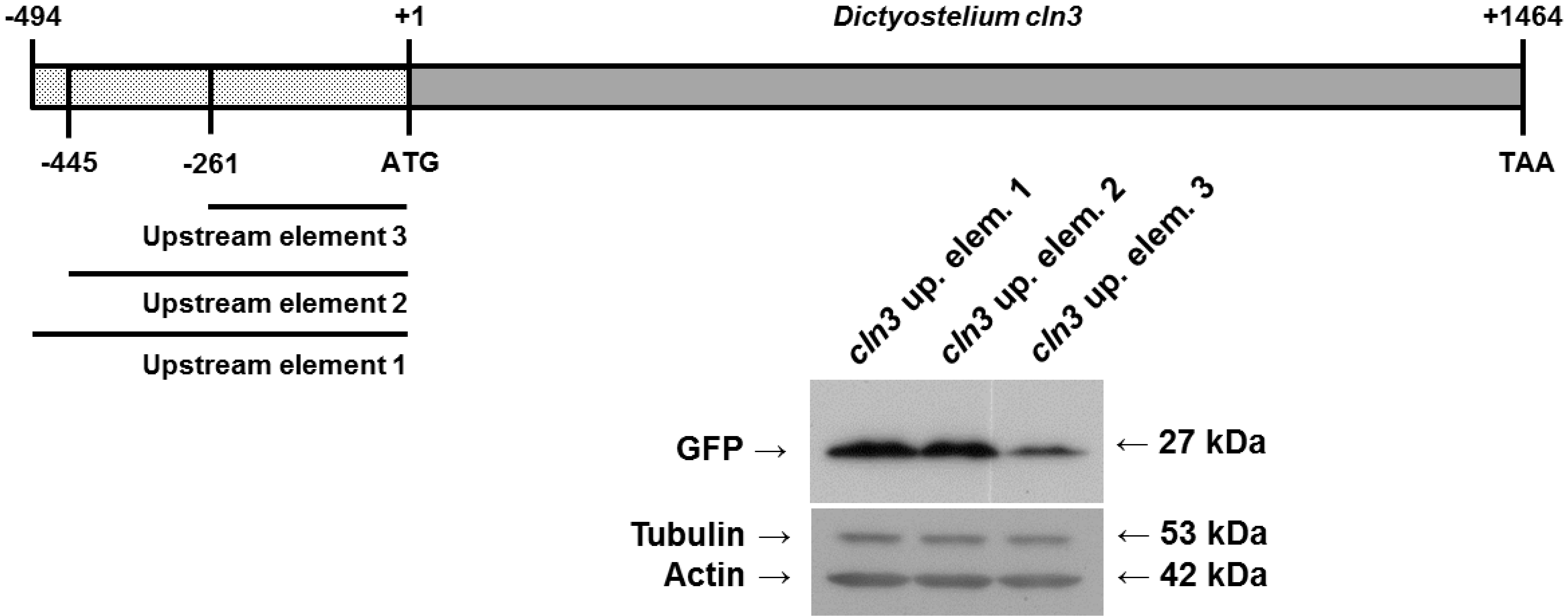

Supplement: Figure S1 — Analysis of gene expression driven by endogenous cln3 upstream elements. AX3 cells were transformed with the appropriate construct (pTX-GFP; act15 promoter replaced with cln3 upstream element 1, 2, or 3) and grown in HL5. Cells were harvested and lysed. Proteins (20 µg) were separated by SDS-PAGE and analyzed by western blotting with anti-GFP, anti-tubulin (loading control), or anti-actin (loading control). Molecular weight markers (in kDa) are shown to the right of each blot. (TIF) [file pone.0110544.s001.tif]

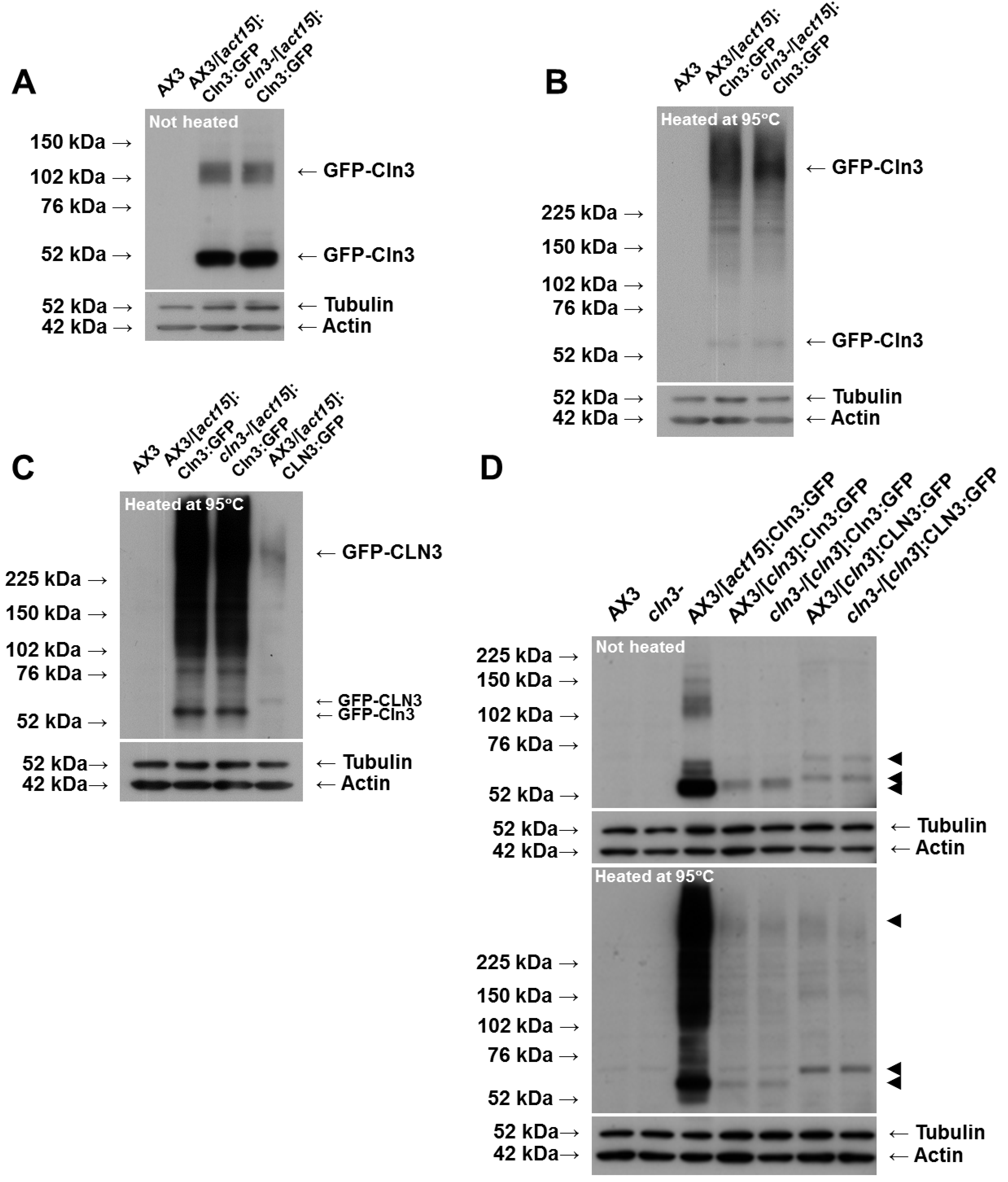

Supplement: Figure S2 — Western blot analysis of Dictyostelium strains expressing Dictyostelium GFP-Cln3 or human GFP-CLN3 under the control of the act15 promoter or cln3 upstream element 1. (A–C) AX3 and cln3− cells were transformed with the appropriate construct (gene expression driven by the act15 promoter) and grown in HL5. Cells were lysed and sample loading buffer was added to whole cell lysates which were either loaded directly into polyacrylamide gels or heated for 5 minutes at 95°C prior to loading into gels. Proteins (20 µg) were separated by SDS-PAGE and analyzed by western blotting with anti-GFP, anti-tubulin (loading control), or anti-actin (loading control). (D) AX3 and cln3− cells were transformed with the appropriate construct (gene expression driven by cln3 upstream element 1) and grown in HL5. Cells were lysed and samples were prepared and analyzed as described above. Molecular weight markers (in kDa) are shown to the left of each blot. (TIF) [file pone.0110544.s002.tif]
