# Supplementary material for: Loss of Cln3 Function in the Social Amoeba Dictyostelium discoideum Causes Pleiotropic Effects That Are Rescued by Human CLN3
Source: PLoS One. 2014 Oct 17;9(10):e110544. doi: 10.1371/journal.pone.0110544 (PMC4201555; doi:10.1371/journal.pone.0110544)
Supplement: Table S1 — List of primers used for cln3 knockout validation and amplification of cln3 upstream elements. The following primers were designed to amplify gDNA from AX3 and cln3− cells to validate the knockout of the cln3 gene in the bsr resistant clone and to amplify fragments upstream of the cln3 start site. The Dictyostelium gene denoted DDB_G0291155 lies downstream of cln3 and was amplified to confirm that the insertion of the bsr cassette did not affect gene DDB_G0291155. (DOCX) [file pone.0110544.s004.docx]

| **Table S1. List of primers used for *cln3* knockout validation and amplification of *cln3* upstream elements.** The following primers were designed to amplify gDNA from AX3 and *cln3^-^* cells to validate the knockout of the *cln3* gene in the *bsr* resistant clone and to amplify fragments upstream of the *cln3* start site. The *Dictyostelium* gene denoted DDB_G0291155 lies downstream of *cln3* and was amplified to confirm that the insertion of the *bsr* cassette did not affect gene DDB_G0291155. | | |  |
| --- | --- | --- | --- |
| **Name** | **Sequence** | **Use** | **Figure** |
| Primer I and VI | AATTTTTCATATTGCGTTGTAAATGC | Knockout mutant validation | 3B |
| Primer II | ATTTATGTCAATTGGTTTAATTGGTG | Knockout mutant validation | 3B |
| Primer III | ACATCATATAAAAGATTGTTGAATTCG | Knockout mutant validation | 3B |
| Primer IV and XI | ACATATTCAAAGAAATAAACCAATGC | Knockout mutant validation | 3B |
| Primer V | ACCACGAAATCCTAATCGAATTTTGGTC | Knockout mutant validation | 3B |
| Primer VII | TTCAAATAATAATTAACCAACCCAAG | Knockout mutant validation | 3B |
| Primer VIII | ATGGATCAATTTAACATTTCTCAAC | Knockout mutant validation | 3B |
| Primer IX | TTAATTTCGGGTATATTTGAGTGG | Knockout mutant validation | 3B |
| Primer X | GAAAATCAAAAAGATAAAGCTGACCCG | Knockout mutant validation | 3B |
| Primer XII | GATGACGAATCAGTTTTAGGAACCTCAG | Knockout mutant validation | 3B |
| DDB_G0291155_F | ATGAATCCAGATTTCACAGTTTATG | Knockout mutant validation; Gene downstream from *cln3* | 3B |
| DDB_G0291155_R | CTTGTCCAACACTATAACCTGCTGC | Knockout mutant validation; Gene downstream from *cln3* | 3B |
| cln3_up_elem_F1 | CACCGTCGACTACAATAAAATAAAATAAATAAATAAAAAAAAT | Forward primer for amplifying *cln3* upstream element 1 | S1 |
| cln3_up_elem_F2 | CAAAGTCGACCAACCCCCTTTTAAAATTTTAC | Forward primer for amplifying *cln3* upstream element 2 | S1 |
| cln3_up_elem_F3 | CAAAGTCGACGAGAAAAAGAGTTTGAATTTTC | Forward primer for amplifying *cln3* upstream element 3 | S1 |
| cln3_up_elem_R1 | CACAGGTACCAATCCAATTTCTAATAAATGTATAATCC | Reverse primer for amplifying *cln3* upstream elements | S1 |
