# Supplementary material for: Loss of Cln3 Function in the Social Amoeba Dictyostelium discoideum Causes Pleiotropic Effects That Are Rescued by Human CLN3
Source: PLoS One. 2014 Oct 17;9(10):e110544. doi: 10.1371/journal.pone.0110544 (PMC4201555; doi:10.1371/journal.pone.0110544)
Supplement: File S1 — Results, Discussion, and References specific to the Supplemental Table and Figures. (DOCX) [file pone.0110544.s005.docx]

**Supporting Information**

**Results**

**Analysis of gene expression driven by endogenous *cln3* upstream elements**

To facilitate the expression of *Dictyostelium* GFP-Cln3 and human GFP-CLN3 at close to endogenous levels, the *act15* promoter and the first 11 codons of the GFP open reading frame, which contained the initiation methionine and an amino-terminal 8x histidine tag, were removed from pTX-GFP and replaced with one of three *cln3* upstream elements. The longest fragment (i.e., upstream element 1) spanned the entire region upstream of the *cln3* start site up to the end of the preceding gene (Fig. S1). The other two fragments (i.e., upstream elements 2 and 3) spanned regions within upstream element 1 up to the *cln3* start site. The three upstream elements, which also included the first 36 base pairs (12 codons) of the *cln3* open reading frame, were then separately cloned into pTX-GFP upstream and in-frame with the GFP open reading frame. The ability of each *cln3* upstream element to drive GFP expression in AX3 cells was verified by western blotting (Fig. S1).

**Generation of *Dictyostelium* strains over-expressing *Dictyostelium* GFP-Cln3 or human GFP-CLN3**

Cells were lysed and proteins from whole cell lysates were separated by SDS-PAGE and analyzed by western blotting with anti-GFP. Results reported in the literature as well as our own experience with other models of JNCL, have shown a propensity of CLN3 to aggregate and precipitate out of solution when heated. Thus in our study, samples were either loaded directly into polyacrylamide gels or heated for 5 minutes at 95°C prior to loading into gels. Anti-GFP detected a strong band of ~50 kDa and a smear between ~100-125 kDa on blots containing unheated samples of AX3 and *cln3^-^* cells transformed with the vector encoding *Dictyostelium* GFP-Cln3 (Fig. S2A). These signals were not observed in AX3 whole cell lysates. On blots containing heated samples, anti-GFP weakly detected a ~60 kDa protein and strongly detected a large smear above 225 kDa (Fig. S2B). AX3 cells were also transformed with a construct encoding human GFP-CLN3. When analyzed by western blotting, anti-GFP weakly detected a ~65 kDa protein and a smear above 225 kDa (Fig. S2C). The ~65 kDa protein was ~5 kDa larger than the band detected in lysates containing *Dictyostelium* GFP-Cln3, which was consistent with their predicted molecular weights. Although anti-GFP detected human GFP-CLN3 in a banding pattern that was similar to *Dictyostelium* GFP-Cln3, the expression of human GFP-CLN3 in AX3 and *cln3^-^* cells was reduced compared to *Dictyostelium* GFP-Cln3 (Fig. S2C). As a result of these findings, an alternative approach was used to express human GFP-CLN3 in *Dictyostelium* cells (Fig. S2D; discussed in Materials and Methods of main text).

**Generation of *Dictyostelium* strains expressing *Dictyostelium* GFP-Cln3 or human GFP-CLN3 under the control of *cln3* upstream element 1**

In unheated whole cell lysates, *Dictyostelium* GFP-Cln3 was detected as a ~55 kDa band while human GFP-CLN3 was detected as a doublet between ~60-70 kDa (Fig. S2D). In samples heated at 95°C for 5 minutes, anti-GFP detected *Dictyostelium* GFP-Cln3 as a doublet between ~60-65 kDa and human GFP-CLN3 as a ~65 kDa protein in AX3 and *cln3^-^* cells (Fig. S2D). Both *Dictyostelium* GFP-Cln3 and human GFP-CLN3 were also detected as a smear above 225 kDa in heated samples (Fig. S2D). When expression was driven by *cln3* upstream element 1, the banding patterns observed for *Dictyostelium* GFP-Cln3 and human GFP-CLN3 were consistent with those observed in the overexpressing strains (Fig. S1,S2). However the levels of expression were reduced, which was consistent with the fact that *cln3* upstream element 1 was driving gene expression in these constructs.

**Discussion**

We detected the *Dictyostelium* GFP-Cln3 fusion protein as a ~50 kDa band on western blots. Although the expected molecular weight of *Dictyostelium* GFP-Cln3 was ~75 kDa (Cln3, 47 kDa, www.dictybase.org; GFP, 27 kDa), the relative hydrophobicity of the protein and evidence from other systems where similar migration characteristics have been observed, support the detection of the fusion protein at a smaller molecular weight. We also detected *Dictyostelium* GFP-Cln3 as a smear between ~100-125 kDa on western blots. In humans, CLN3 is heavily glycosylated causing it to be detected at a significantly higher than expected molecular weight [1]. The sites of glycosylation in CLN3 are conserved in the *Dictyostelium* ortholog indicating that Cln3 is also likely glycosylated. In humans, CLN3 is also phosphorylated, palmitoylated, and prenylated and these post-translational modifications could have also affected the migration rates of the fusion proteins in this study [2-8]. When samples were heated prior to loading into gels, the Cln3 GFP-fusion proteins were detected by western blotting as large smears above 225 kDa, which can be explained by the tendency of Cln3 to aggregate when heated. Taken together, these results indicate that *Dictyostelium* Cln3 protein behaves similarly to CLN3 from other systems when analyzed by western blotting.

**References**

[1] Ezaki J, Takeda-Ezaki M, Koike M, Ohsawa Y, Taka H, et al (2003) Characterization of Cln3p, the gene product responsible for juvenile neuronal ceroid lipofuscinosis, as a lysosomal integral membrane glycoprotein. J Neurochem 87:1296-1308.

[2] Michalewski MP, Kaczmarski W, Golabek AA, Kida E, Kaczmarski A, et al (1998) Evidence for phosphorylation of CLN3 protein associated with Batten disease. Biochem Biophys Res Commun 253:458-462.

[3] Michalewski MP, Kaczmarski W, Golabek AA, Kida E, Kaczmarski A, et al (1999) Posttranslational modification of CLN3 protein and its possible functional implication. Mol Genet Metab 66:272-276.

[4] Kaczmarski W, Wisniewski KE, Golabek A, Kaczmarski A, Kida E, et al (1999) Studies of membrane association of CLN3 protein. Mol Genet Metab 66:261-264.

[5] Järvelä I, Sainio M, Rantamaki T, Olkkonen VM, Carpén O, et al (1998) Biosynthesis and intracellular targeting of the CLN3 protein defective in Batten disease. Hum Mol Genet 7:85-90.

[6] Kida E, Kaczmarski W, Golabek A, Kaczmarski A, Michalewski M, et al (1999) Analysis of intracellular distribution and trafficking of the CLN3 protein in fusion with the green fluorescent protein in vitro. Mol Genet Metab 66:265–271.

[7] Pullarkat RK, Morris GN (1997) Farnesylation of Batten disease CLN3 protein. Neuroped 28:42-44.

[8] Storch S, Pohl S, Quitsch A, Falley K, Braulke T (2007) C-terminal prenylation of the CLN3 membrane glycoprotein is required for efficient endosomal sorting to lysosomes. Traffic 8:431–444.
